# Immune Regulation and ECM-Related Pathway Enrichment Reveal ATP2A3 as a Prognostic Biomarker for Nonspecific Orbital Inflammation: An Integrated Machine Learning and Mendelian Randomization Analysis

**DOI:** 10.1155/mi/7061507

**Published:** 2025-06-13

**Authors:** Zixuan Wu, Xi Long, Kang Tan, Xiaolei Yao, Qinghua Peng

**Affiliations:** ^1^Hunan University of Traditional Chinese Medicine, Changsha 410208, Hunan Province, China; ^2^Department of Ophthalmology, The First Affiliated Hospital of Hunan University of Traditional Chinese Medicine, Changsha 410007, Hunan Province, China

**Keywords:** ATP2A3, autoimmune inflammatory disorder, Lasso regression, Mendelian randomization, nonspecific orbital inflammation (NSOI)

## Abstract

**Background:** Nonspecific orbital inflammation (NSOI) is a heterogeneous inflammatory disorder of the orbit with an unclear etiology. ATP2A3, a key regulator of calcium homeostasis in the endoplasmic reticulum (ER), may play a pivotal role in NSOI pathogenesis. Its potential as a diagnostic biomarker merits thorough investigation.

**Methods:** Differentially expressed genes (DEGs) common to two GEO datasets (GSE58331 and GSE105149) were intersected with immune-related genes from the ImmPort database, yielding 89 candidates. ATP2A3 was prioritized using machine learning (ML) approaches, including LASSO, support vector machine (SVM)-RFE, and weighted gene coexpression network analysis (WGCNA). Functional enrichment was assessed using GSEA and GSVA based on genes co-expressed with ATP2A3. Immune microenvironment characteristics were evaluated using CIBERSORT and ESTIMATE. Expression of ATP2A3 was validated in GSE105149.

**Results:** Fifteen hub genes were identified, with ATP2A3 strongly linked to immune-related pathways. Genes positively correlated with ATP2A3 were enriched in sensory perception and extracellular matrix (ECM) organization. Immune infiltration analysis revealed a positive association between ATP2A3 expression and memory B cells, M2 macrophages, resting mast cells, monocytes, and regulatory T cells (Tregs), while naive B cells and plasma cells were negatively associated. ATP2A3 exhibited significant diagnostic potential for distinguishing NSOI.

**Conclusions:** In the context of NSOI, we identify ATP2A3 as a novel contributor to immune-driven pathogenesis. Its significant dysregulation in NSOI tissues relative to healthy controls underscores its potential as a prognostic marker within the inflammatory microenvironment.

## 1. Introduction

Nonspecific orbital inflammation (NSOI) is a benign and noninfectious inflammatory condition that is confined to the orbit and is characterized by the absence of identifiable systemic or localized etiological triggers. Representing approximately 6%–16% of all orbital disorders and 11% of orbital neoplastic-like presentations, NSOI shows a notable predilection for middle-aged individuals, with a higher incidence observed in females [1, 2]. Clinically, NSOI presents with a spectrum of symptoms—including periorbital swelling, pain, erythema, and variable degrees of proptosis—depending on the anatomical site of involvement. Inflammation may affect the extraocular muscles, lacrimal gland, or orbital fat. In more advanced cases, extraocular muscle involvement can lead to ophthalmoplegia and diplopia, while optic nerve compression may compromise visual acuity [3, 4]. Importantly, the clinical manifestations of NSOI can closely mimic those of other orbital pathologies, such as thyroid-associated orbitopathy, lymphoproliferative disorders, and orbital neoplasms, often complicating the diagnostic process [5]. Radiological investigations, particularly magnetic resonance imaging (MRI) and computed tomography (CT), typically reveal ill-defined and infiltrative lesions or localized tissue thickening. While these modalities provide critical anatomical detail, histopathological confirmation remains the gold standard, particularly when malignancy or systemic disease cannot be definitively ruled out [6]. Histology frequently reveals a polymorphous inflammatory infiltrate composed of lymphocytes, plasma cells, eosinophils, and macrophages, reflecting active immune engagement. Despite its well-documented clinical and histological features, the underlying pathogenesis of NSOI remains poorly defined [7]. The absence of consistent infectious or autoimmune etiologies has led to the prevailing hypothesis that NSOI may arise from a dysregulated immune response, potentially influenced by genetic or environmental factors. However, definitive molecular mechanisms remain elusive [8]. In this context, a deeper understanding of the molecular and immunological pathways driving NSOI is urgently needed. Elucidating these pathways could yield novel insights into disease initiation and progression, while identifying actionable targets for therapeutic intervention [9]. Molecular dissection of NSOI through transcriptomic and bioinformatic approaches may ultimately inform the development of targeted therapies, reduce recurrence rates and improve clinical outcomes for affected individuals.

ATP2A3, which encodes the sarco/endoplasmic reticulum Ca^2+^-ATPase 3 (SERCA3), is a key mediator of calcium homeostasis in the endoplasmic reticulum (ER), essential for maintaining intracellular calcium dynamics [10]. As a member of the P-type ATPase family, SERCA3 facilitates the active transport of calcium ions from the cytosol into the ER lumen, a process fundamental to diverse cellular functions including protein folding, signal transduction, and apoptosis [11]. Although ATP2A3 is broadly expressed, its activity exhibits tissue specificity, with pronounced roles in nonmuscle tissues such as the immune system, gastrointestinal tract, and pancreatic β-cells [12]. Aberrant expression or functional impairment of ATP2A3 has been implicated in a range of pathologies, particularly those driven by disrupted calcium signaling [13]. In cancer, downregulation of ATP2A3 has been reported across multiple tumor types. This reduction may disturb calcium-dependent signaling networks, thereby promoting oncogenic pathways, supporting uncontrolled proliferation and enabling apoptosis resistance [14]. Moreover, impaired ATP2A3 function has been linked to chemoresistance, suggesting that restoring its expression or activity could offer therapeutic benefit. Beyond oncology, ATP2A3 has emerged as a regulator of immune responses [15]. Dysregulated expression in immune cells may contribute to abnormal activation and inflammatory cascades, with potential implications in autoimmune disease. These dual roles—governing both tumor biology and immune homeostasis—underscore ATP2A3 as a promising therapeutic target [16]. In the context of ocular inflammation, particularly NSOI, the ability to modulate immune responses locally or systemically offers an attractive strategy for intervention. Through regulation of calcium flux within the ER, ATP2A3 orchestrates essential cellular functions, including proliferation, apoptosis, and immune signaling. Increasing evidence links calcium dysregulation to aberrant inflammatory responses, positioning ATP2A3 as a potential mediator of immune-driven pathologies. In this context, ATP2A3 emerges as a compelling candidate for elucidating the pathogenesis of NSOI and as a prospective biomarker for its diagnosis and progression monitoring.

**Figure 1:**
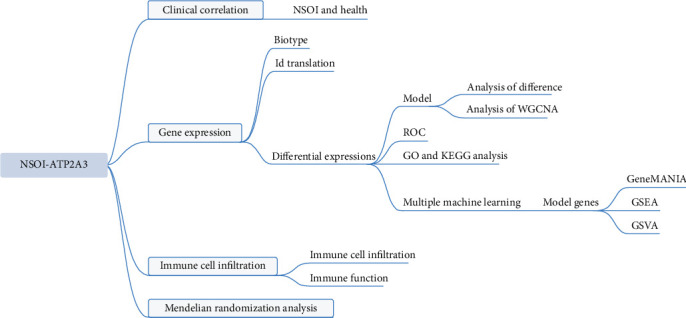
Framework.

The integration of high-throughput transcriptomic technologies with richly annotated clinical datasets from the NSOI initiative has opened new avenues for unraveling the transcriptional architecture and molecular circuitry of NSOI [7, 17, 18]. Machine learning (ML), a core domain within artificial intelligence, offers powerful data-driven algorithms capable of uncovering hidden patterns and enhancing predictive accuracy without explicit programming [19]. Among the molecular processes implicated in orbital diseases, calcium signaling plays a central role in maintaining cellular homeostasis. In orbital tissues, calcium ions act as critical secondary messengers, regulating key physiological functions including proliferation, apoptosis, differentiation, and immune responses. Perturbations in calcium flux—often resulting from dysregulation of calcium transporters or channels—can destabilize tissue homeostasis, leading to chronic inflammation and fibrotic remodeling. In NSOI, the convergence of aberrant calcium signaling and immune activation is particularly pronounced. Disrupted intracellular calcium dynamics can potentiate pro-inflammatory signaling, alter immune cell activity, and skew cytokine profiles, thereby contributing to disease onset and progression. Furthermore, calcium-dependent pathways are instrumental in modulating the behavior of orbital fibroblasts, macrophages, and other immune effectors in response to environmental or autoimmune stimuli. Defining the specific roles of calcium-regulatory proteins in NSOI could offer novel insights into disease mechanisms and guide the development of targeted interventions. The application of advanced bioinformatics to high-throughput datasets has emerged as a critical strategy for delineating functional gene networks and uncovering molecular mechanisms in complex diseases [20, 21]. This study seeks to address this gap through an in-depth analysis of NSOI-related GEO datasets, aiming to uncover key molecular players and biological pathways implicated in disease pathogenesis (Figure 1).

## 2. Materials and Methods

We used the approaches proposed by Wu et al. [22].

### 2.1. Transcriptional Profiling Data and Determination of Differentially Expressed Genes (DEGs)

**Figure 2:**
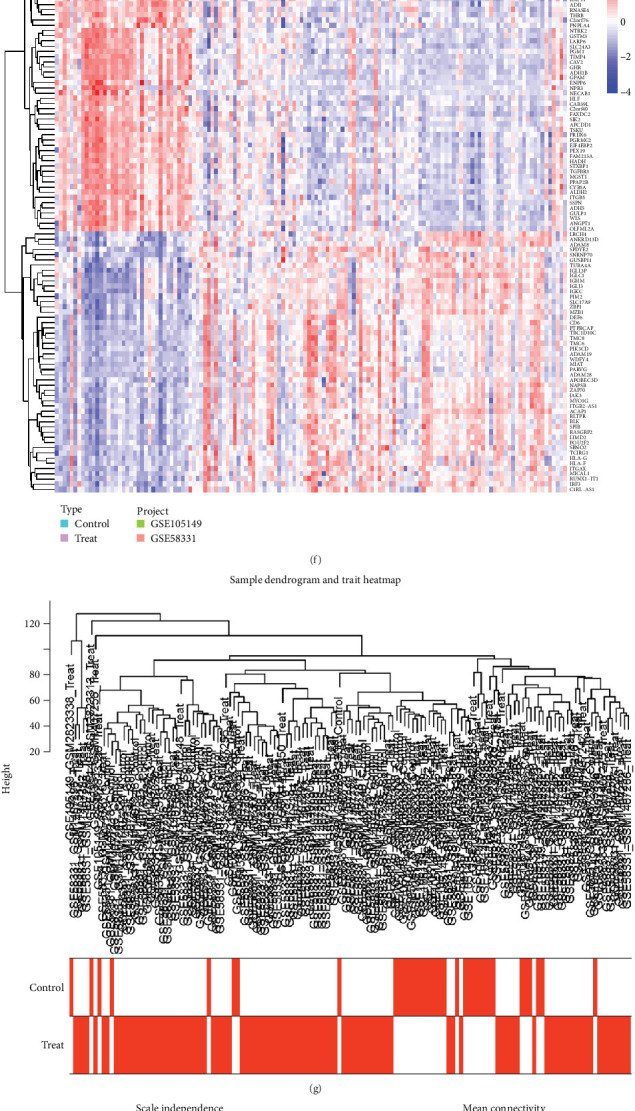
Principal component analysis and model. (a–d) PCA results. *Note*: Red is GSE1-5149; green is GSE58331. (e) Heatmap of DEGs. (f) Volcano plot of DEGs. (g, h) Soft threshold power mean connection and scale-free fitting index analysis. (i) Clustering of dendrograms. (j, k) Heatmap of correlations between module eigengenes and clinical characteristics. *Note*: In type, purple is the expression in NSOI, and light blue is the expression in control. In the vertical axis, red indicates high expression, blue indicates low expression, and the darker the color, the more significant the expression. (l) Gene scatterplot in the blue module.

mRNA expression datasets relevant to NSOI were sourced from the GEO. Specifically, this study analyzed datasets GSE58331 and GSE105149, both derived from the GPL570-55999 platform (Table S1). For genes associated with multiple probes, the arithmetic mean of the probe values was calculated to represent the gene's overall expression level. Data correction for multichip analyses was performed using the “Sva” and “Limma” packages in R for batch normalization. Following dataset standardization, batch effect correction was conducted utilizing the SVA package and the effectiveness of this adjustment was assessed via PCA (Figure 2a–d). From the PCA plot, it can be seen that this dataset can divide the data well. Differential gene expression analysis between NSOI and control groups was performed using the limma package in R. For genes represented by multiple probes, expression values were aggregated using the arithmetic mean to derive a single representative expression level per gene. DEGs were identified based on the threshold of an absolute log_2_ fold change (|log_2_ FC|) greater than 1 and an adjusted *p*-value <0.05. The analysis prioritized DEGs with potential roles in immune infiltration, given their relevance to the immunopathogenesis of NSOI.

### 2.2. Weighted Gene Coexpression Network Analysis (WGCNA)

Following data preprocessing, coexpression network construction was performed using the GSE58331 and GSE105149 transcriptomic datasets. Soft-thresholding powers of *β* = 20 and *β* = 9 were selected for GSE58331 and GSE105149, respectively, in accordance with the standard WGCNA protocol to ensure biologically meaningful scale-free network topology. The selection criteria were threefold: (i) achieving a scale-free topology fit index (*R*^2^) > 0.85, (ii) maintaining adequate mean connectivity, and (iii) preserving network sparsity to reduce noise from low-confidence correlations. For GSE105149, *β* = 9 was the minimum power yielding a high scale-free topology fit (*R*^2^ > 0.85) while retaining strong average connectivity. Similarly, for GSE58331, *β* = 20 achieved an optimal balance between scale independence and network connectivity. These threshold values ensured the robustness of downstream module detection and functional interpretation.

Coexpression modules were first delineated using a dynamic tree-cutting algorithm, followed by eigengene-based hierarchical clustering to consolidate highly similar modules. A merging threshold of 0.25—corresponding to a Pearson correlation coefficient of 0.75 between module eigengenes—was applied to reduce redundancy and enhance biological interpretability. This cutoff, commonly employed in WGCNA literature, balances module specificity with the integration of functionally related gene sets. Based on this criterion, gene modules exhibiting strong intermodule connectivity were merged, yielding a total of 10 distinct modules. Among these, the blue module exhibited the most robust correlation with the trait of interest. DEGs were subsequently intersected with genes from the identified modules for downstream analysis.

### 2.3. Functional Enrichment Analysis and Multiple ML

We conducted a comprehensive functional enrichment analysis to decipher the biological significance of DEGs. Enriched terms were clustered based on semantic similarity, and the most representative term from each cluster was selected for downstream interpretation. To further delineate the biological functions and signaling pathways associated with DEGs, GO, and KEGG enrichment analyses were performed using the clusterProfiler package (v4.0) in R, with statistical significance defined as *p*  < 0.05. To construct a robust and predictive molecular signature, we implemented an integrative ML framework comprising 22 algorithms and 89 combinatorial models. These included regularization-based methods such as Lasso, Ridge, and Elastic Net (Enet, with *α* ranging from 0.1 to 0.9), classical approaches like stepwise generalized linear models (StepGLM; both forward and backward directions), support vector machines (SVMs), and advanced ensemble techniques including random forest (RF), generalized boosted models (GBMs), XGBoost, and Naive Bayes. Additional dimension reduction and classification strategies—such as partial least squares regression generalized linear model (plsRglm) and linear discriminant analysis (LDA)—were also incorporated. Model performance and generalizability were rigorously assessed using two independent validation cohorts: GSE58331 and GSE105149. This multialgorithmic approach enabled the identification of a consensus gene signature with high predictive accuracy and cross-dataset robustness.

### 2.4. GSEA and GSVA

To unravel the mechanisms underlying these DEGs, we constructed an extensive gene regulatory network using GeneMANIA (https://genemania.org/). To further explore the functional dynamics and pathway alterations across diverse samples, we employed GSEA and GSVA. These robust methodologies enabled the identification of functionally related gene sets and highlighted pathway changes through quantitative scores and visual representations, revealing active biological processes and pathways across various risk stratifications. Utilizing R, we conducted an in-depth investigation into the impact of DEGs on BP, MF, CC, and relevant pathways. This comprehensive approach provided a detailed understanding of the roles these genes play in disease mechanisms. By uncovering complex biological themes and molecular pathways influenced by these DEGs, we enhanced our understanding of their involvement in disease pathology.

### 2.5. Immune Infiltration Analysis

We performed a comprehensive analysis of immune cell infiltration between NSOI and normal tissue samples from the GSE58331 dataset, as well as between small and large NSOI samples, utilizing the CIBERSORT algorithm with the parameter “PERM” set to 1000 and a significance threshold of *p*  < 0.05. The relative proportions of each immune cell type were quantified and visualized via bar plots. To further explore the immune landscape, we employed the “pheatmap” package to generate a heatmap depicting the abundance of 22 distinct immune cell populations, while violin plots, created using the “vioplot” package, illustrated the distribution of cell types across samples. Correlation between these immune cell subsets was assessed and visualized through a correlation heatmap generated using the “corrplot” package, highlighting the interrelationships among the 22 infiltrating immune cell types.

### 2.6. Establishing Causality via Mendelian Randomization (MR)

To rigorously investigate the putative causal relationship between genetic predisposition and the incidence of NSOI, we performed a MR analysis using the TwoSampleMR package in R. Specifically, we assessed the causal impact of ATP2A3 gene expression—designated as the exposure—on NSOI, the outcome of interest, drawing summary statistics from publicly available GWAS datasets (https://gwas.mrcieu.ac.uk/). (1) Instrumental variable (IV) selection: Single nucleotide polymorphisms (SNPs) strongly associated with ATP2A3 expression were selected as candidate IVs based on a genome wide significance threshold (*p*  < 5 × 10^−8^), ensuring the relevance of the instruments to the exposure. (2) IV independence: To eliminate linkage disequilibrium (LD) and maintain independence among instruments, we applied PLINK-based clumping with an LD threshold of *r*^2^ < 0.001 and a physical distance cutoff of 10,000 kb. This step was critical to prevent confounding due to correlated variants and to mitigate horizontal pleiotropy. (3) Instrument strength and validity: Each SNP was further evaluated for strength using the *F*-statistic (*F* = *β*^2^/SE^2^), where *β* denotes the SNP's effect on ATP2A3 expression and SE its standard error. SNPs with *F*  < 10 were excluded to reduce weak instrument bias and ensure sufficient explanatory power, consistent with established MR standards. (4) Sensitivity analyses: To detect and correct for potential violations of core MR assumptions, we conducted a series of sensitivity tests. MR-Egger regression was employed to assess directional pleiotropy, with the intercept term indicating the presence of unbalanced pleiotropic effects. Additionally, the MR-PRESSO (pleiotropy residual sum and outlier) test was performed to identify and correct for outlier SNPs exerting pleiotropic effects via pathways unrelated to the exposure. Together, this multilayered approach—combining rigorous IV selection, LD pruning, instrument strength evaluation, and pleiotropy assessment—ensured the robustness of causal inference and strengthened the validity of ATP2A3 as a potential contributor to NSOI pathogenesis.

## 3. Results

### 3.1. DEG and WGCNA Genes Identification

Differential analyses were performed using the limma package of R4.31 software. With pheatmap package to build the heatmap [23]. We integrated datasets GSE58331 and GSE105149 and performed batch effect correction. PCA confirmed the successful segregation of patients into risk-specific cohorts (Figure 2a–d). Notably, some DEGs were significantly upregulated, whereas others were markedly downregulated (Figure 2e). Among the 1217 DEGs, several exhibited significant differences. Specifically, certain genes clustered within the treatment group (ANKRD13D, ADAM8, SPDYE2, SNRNP70, GUSBP11, TUBA4A, IGLL3P, IGLC1, IGHM, etc.), while others were prevalent in the control group (SLC24A3, PGM1, TIMP4, CAV2, GHR, ADH1B, GPAM, ENPP6, NPR3, NECAB1, HLF, CAB39L, etc.; Figure 2f; Table S2). To approximate a scale-free topology for the network, a soft-thresholding power was applied (Figure 2g,h). Genes with the highest variance were clustered and organized into 10 coexpression modules (Figure 2i). Pearson's correlation analysis was employed to examine the relationship between module eigengenes and clinical characteristics (Figure 2j,k). Among the identified modules, the blue module exhibited the strongest correlation with the “Group” attribute, distinguishing between NSOI and normal samples and showed the highest degree of association (Figure 2l).

### 3.2. Construction of the Model and Enrichment Analysis

**Figure 3:**
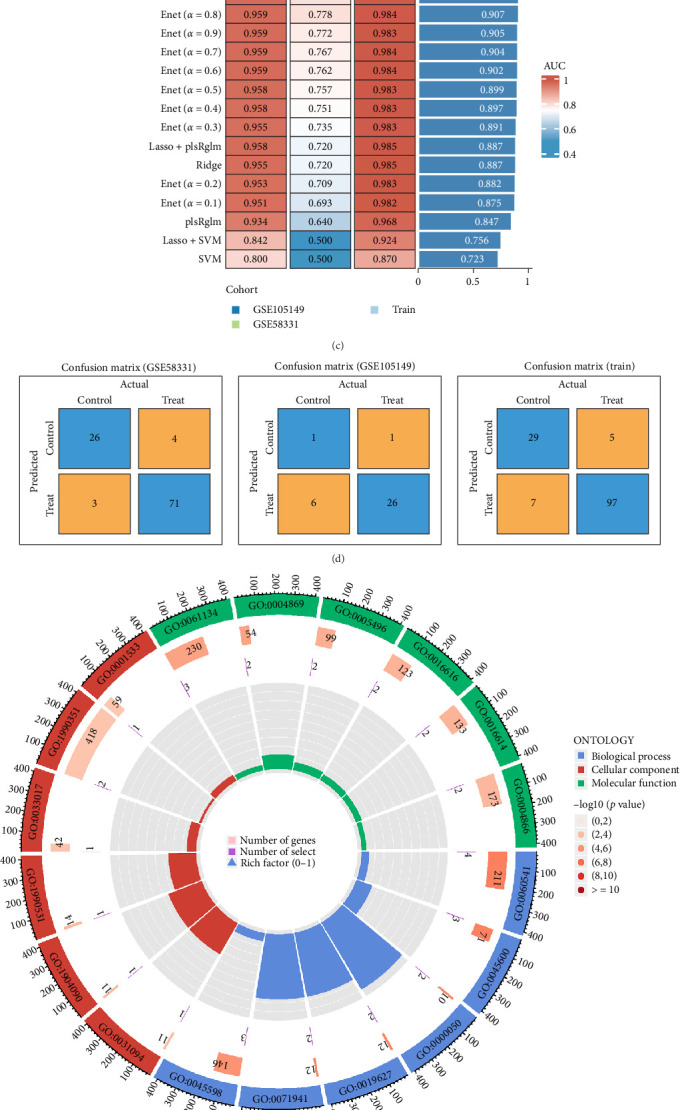
Construction of the model and enrichment analysis. (a) VNN. (b) ROC of the dataset. (c) Machine learning. (d) Validation of dataset. *Note:* Darker color (dark blue, dark red, etc.) indicates that the larger the value in that cell, that is, these kinds of predictions are more frequent. Lighter color indicates a smaller value in that cell. (e) GO analysis. (f) KEGG analysis. *Note:* The longer bar means the more genes enriched, and the increasing depth of red means the differences were more obvious. The GO circle shows the scatter map of the log FC of the specified gene. The higher the *Z*-score value indicated, the higher expression of the enriched pathway.

Attempts to integrate key genes from LASSO and WGCNA provided the most stable model constructions. Ultimately, we identified 15 hub genes (Figure 3a; Table S3). We performed 22 ML on GSE58331 and GSE105149. From the results, this shows that the GSE58331 (AUC: 0.987; 95% CI: 0.970−0.998), GSE105149 (AUC: 0.804; 95% CI: 0.603−0.963), and training datasets (AUC: 0.965; 95% CI: 0.934−0.988) have high stability (Figure 3b). We incorporated 22 ML algorithms and 89 algorithm combinations. Through Figure 3c, we conclude that Stepglm (forward) has the highest accuracy in this analysis (0.965). Therefore, we selected this ML approach for the next analysis. In addition, GSE58331, GSE105149, and training datasets were used to validate the ML results. Confusion matrices are illustrated in GSE58331, GSE105149, and training datasets, respectively. This shows that the results are stable and credible (Figure 3c,d). GO enrichment analysis identified 406 core targets, encompassing biological processes BP, MF, and CC. MF included DNA-binding transcription activator activity (GO:0001216), DNA-binding transcription activator activity, RNA polymerase II-specific (GO:0001228), and metal ion transmembrane transporter activity (GO:0046873). CC included sarcoplasmic reticulum (GO:0016529), neuronal cell body (GO:0043025), and transporter complex (GO:1990351). BP involved lipid localization (GO:0010876), epithelial cell proliferation (GO:0050673), and negative regulation of immune system process (GO:0002683). KEGG enrichment analysis indicated that the overexpressed genes were primarily involved in biosynthesis of amino acids (hsa01230), cysteine and methionine metabolism (hsa00270), and transcriptional misregulation in cancer (hsa05202; Figure 3e,f; Table S4a,b).

### 3.3. Identification of Model Gene

**Figure 4:**
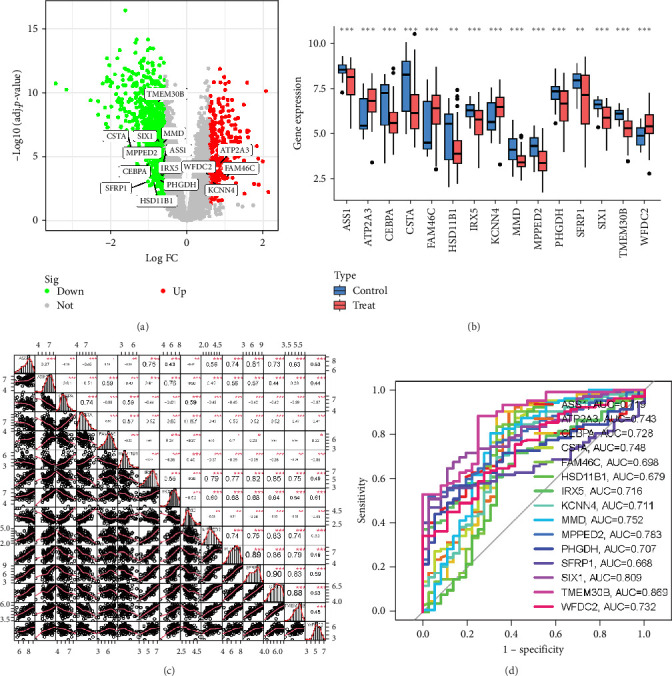
Identification of model gene. (a) Volcano plot. (b, c) Expression of model genes. *Note:* Blue indicates expression in the control group and red indicates expression in the NSOI group. The ordinate indicates the expression content of the gene in the two groups. p Values were showed as: *⁣*^*∗*^p  < 0.05; *⁣*^*∗∗*^p < 0.01; *⁣*^*∗∗∗*^p  < 0.001. (d) AUC of 15 hub genes.

**Figure 5:**
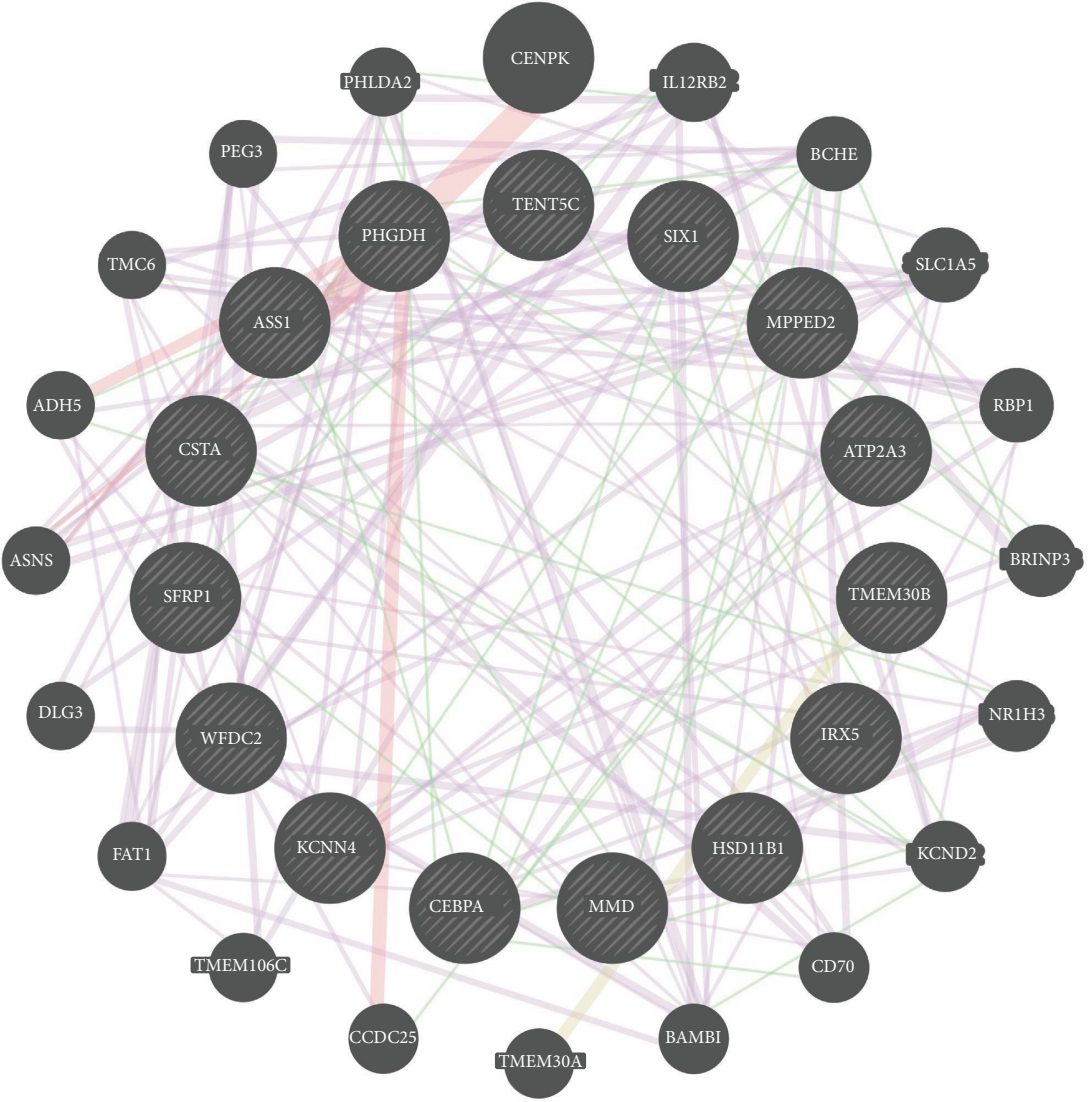
Coexpression network of model genes. *Note:* The red line represents stronger connectivity and the purple is slightly weaker. Thicker lines represent stronger connectivity.

After applying the differential genes to the ML model, 15 DEGs were obtained. Notably, some DEGs were significantly upregulated (ATP2A3, FAM46C, and KCNN4), whereas others were markedly downregulated (TMEM30B, CSTA, SIX1, MMD, MPPED2, ASS1, CEBPA, IRX5, WFDC2, SFRP1, PHGDH, and HSD11B1; Figure 4a). Specifically, we employed Spearman's rank correlation coefficient, a nonparametric measure of monotonic association that is robust to outliers and nonlinear relationships. This information has been clearly annotated in both the figure legend and the Methods section, thereby enhancing the interpretability and reproducibility of the correlation analysis. Specifically, certain genes clustered within the treatment group (ATP2A3, FAM46C, KCNN4, and WFDC2), while others were prevalent in the control group (ASS1, CEBPA, CSTA, IRX5, HSD11B1, MMD, MPPED2, PHGDH, SFRP1, SIX1, and TMEM30B; Figure 4b,c). The ROC analysis of the 15 hub genes further confirmed their high predictive accuracy (AUC): ASS1-0.719, ATP2A3-0.743, CEBPA-0.728, CSTA-0.748, FAM46C-0.698, HSD11B1-0.679, IRX5-0.716, KCNN4-0.711, MMD-0.752, MPPED2-0.783, PHGDH-0.707, SFRP1-0.668, SIX1-0.809, TMEM30B-0.869, and WFDC2-0.732. These results confirmed the high reliability and accuracy of our model (Figure 4d). To elucidate the underlying mechanisms of model genes, we established an extensive gene regulatory network (Figure 5). Within this network, CENPK, IL12RB2, BCHE, SLC1A5, and RBP1 demonstrated robust interactions with model genes, known for its roles in inflammation and immunity. The intricate relationships within the gene regulatory network are detailed in Table S5.

### 3.4. GSEA Analysis of Model Genes

**Figure 6:**
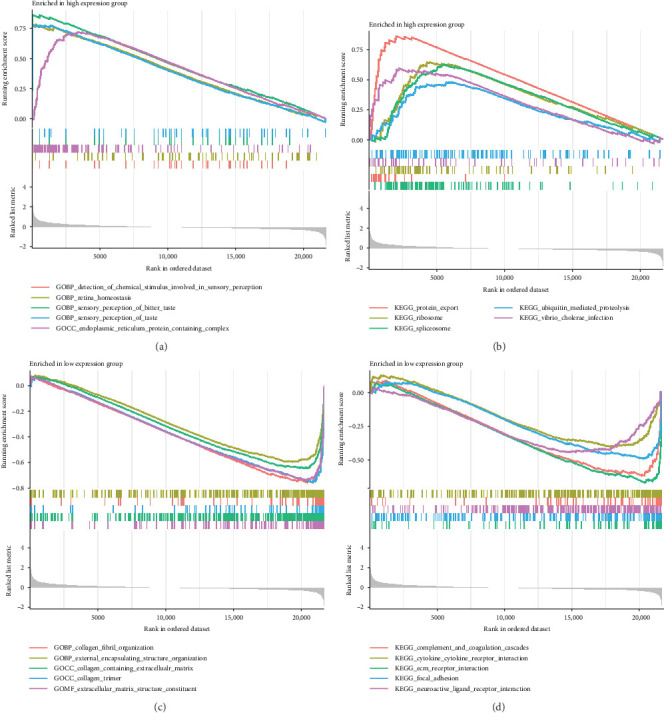
GSEA of ATP2A3. (a, c) GSEA of GO analysis. (b, d) GSEA of KEGG analysis. *⁣*^*∗*^*p* < 0.05; *⁣*^*∗∗*^*p* < 0.01; *⁣*^*∗∗∗*^*p* < 0.001.

To elucidate the functional implications of the model genes in NSOI, GSEA was conducted on DEGs. In the high expression subgroup, GO enrichment revealed a predominance of biological processes related to sensory perception of bitter taste and general taste, as well as molecular functions associated with the ER protein-containing complex. These enrichments suggest a potential, albeit unconventional, link between sensory transduction pathways and the immunopathology of NSOI, possibly reflecting a broader role of chemosensory signaling in immune modulation. Conversely, the low expression group was enriched in biological processes and cellular components involved in the organization of external encapsulating structures and the extracellular matrix (ECM), specifically collagen-containing ECM and collagen trimers (Figure 6a). These findings imply a downregulation of tissue remodeling and structural integrity maintenance, processes intimately associated with fibrosis and chronic inflammation in the orbital microenvironment. KEGG pathway analysis further corroborated these insights. In the high expression group, enrichment of pathways such as protein export, ribosome, and spliceosome indicates an upregulation of translational and posttranscriptional machinery, potentially reflective of heightened biosynthetic activity in inflamed tissues. In contrast, the low expression group exhibited significant enrichment in immune- and ECM-related pathways, including cytokine–cytokine receptor interaction, ECM-receptor interaction, and focal adhesion—hallmarks of chronic inflammatory responses and tissue remodeling characteristic of NSOI. Together, these results underscore the dual contributions of immune dysregulation and ECM alterations to the pathogenesis of NSOI, while also hinting at novel molecular processes that may modulate disease progression (Figure 6b; Table S6).

### 3.5. GSVA Analysis of Model Genes

**Figure 7:**
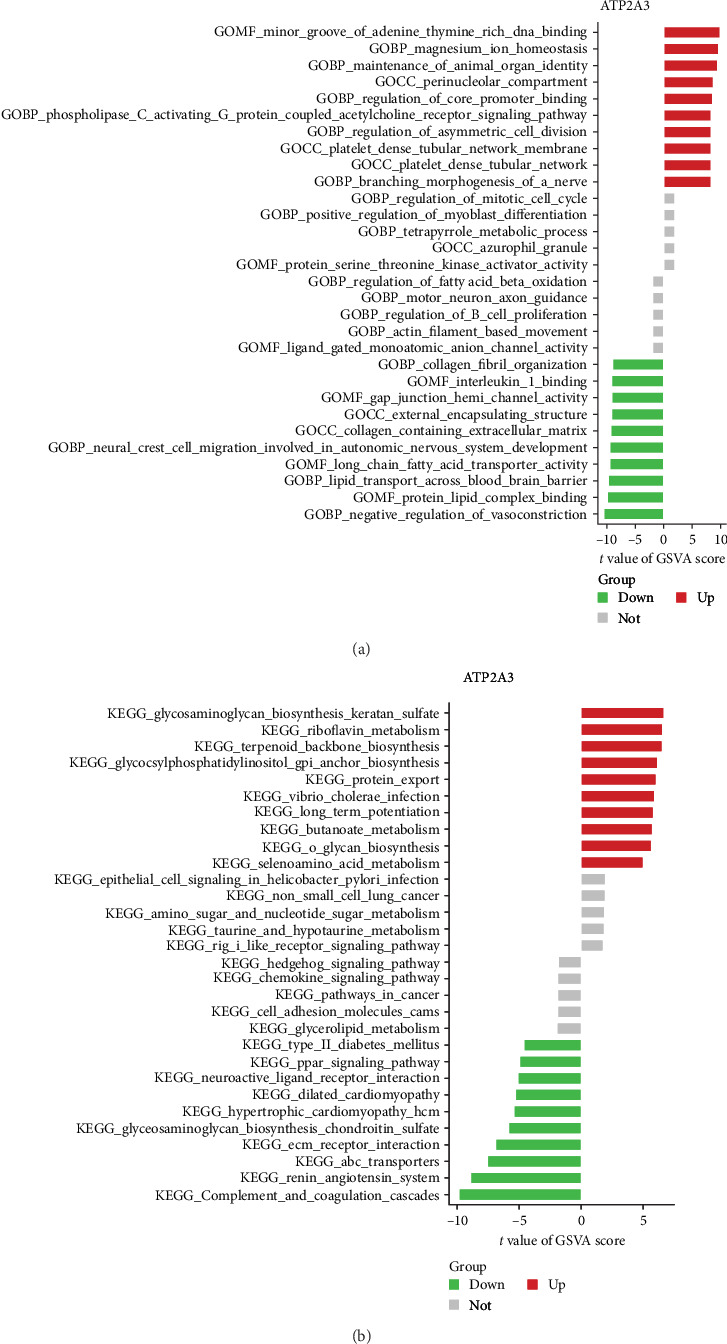
GSVA of ATP2A3. (a) GSVA of GO analysis. (b) GSVA of KEGG analysis.

To gain deeper insights into the biological processes associated with the model gene in NSOI, GSVA was employed. In the GO enrichment analysis, the high expression phenotype was characterized by upregulation of gene sets involved in the regulation of core promoter binding, the perinucleolar compartment, maintenance of animal organ identity, and magnesium ion homeostasis, alongside molecular functions related to minor groove binding of adenine–thymine-rich DNA. These findings point toward a potential role for transcriptional regulation and chromatin organization in shaping the inflammatory transcriptional landscape of NSOI. The enrichment of magnesium ion homeostasis may further reflect perturbations in ion-mediated signaling and cellular stress responses, both of which are increasingly recognized in chronic inflammatory milieus (Figure 7a). Complementary KEGG pathway analysis revealed enrichment of metabolic and biosynthetic pathways, including protein export, glycosylphosphatidylinositol (GPI)-anchor biosynthesis, terpenoid backbone biosynthesis, riboflavin metabolism, and glycosaminoglycan biosynthesis—specifically keratan sulfate. Notably, GPI-anchor biosynthesis and glycosaminoglycan metabolism are integral to membrane protein anchoring and ECM dynamics, respectively, suggesting a possible mechanistic link between metabolic reprogramming and tissue remodeling in NSOI. The upregulation of these biosynthetic pathways may reflect the heightened cellular turnover and matrix reorganization observed in inflamed orbital tissues. Collectively, these data suggest that NSOI pathogenesis may be shaped not only by classical immune dysregulation but also by transcriptional reprogramming and metabolic adaptation, which together contribute to the structural and functional perturbations of the orbital microenvironment (Figure 7b).

### 3.6. Immune Landscape Characterization

**Figure 8:**
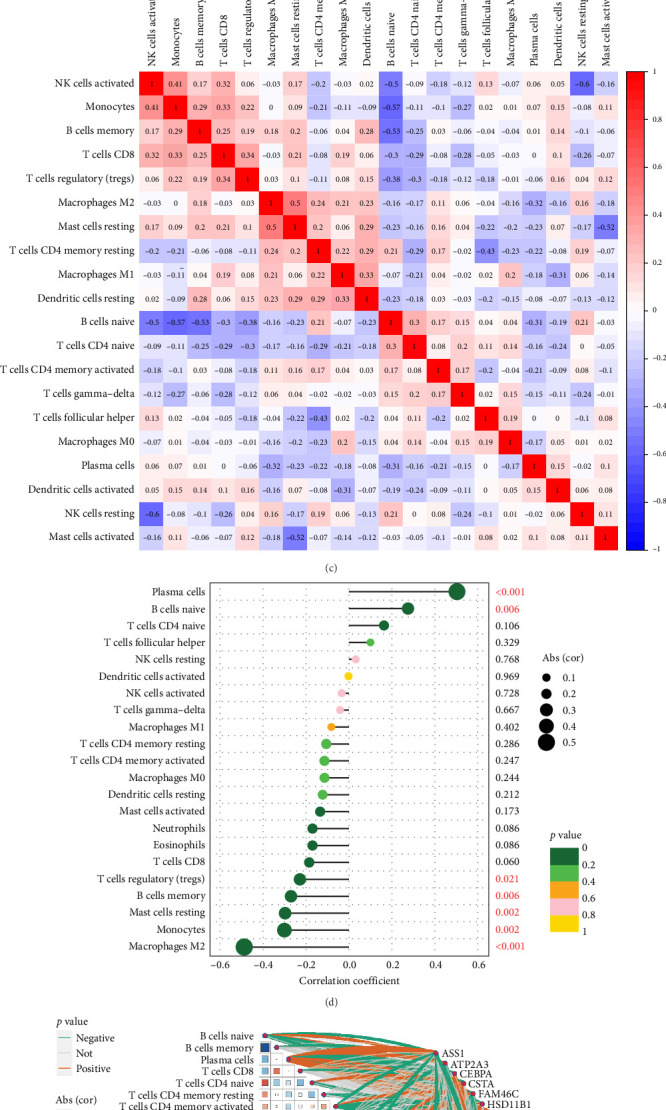
Immune landscape characterization. (a) Immune function and cells. (b, c) Correlation rectangle plot. (Red line: positive correlation; blue line: negative correlation. The depth of the colors reflects the strength of the relevance). (d) Lollipop plot. (e) LinkET plot. (f) Immune infiltration analyses. *⁣*^*∗*^*p* < 0.05; *⁣*^*∗∗*^*p* < 0.01; *⁣*^*∗∗∗*^*p* < 0.001.

The immunological environment plays a crucial role in the initiation and progression of NSOI. Notably, the risk-associated profiles revealed substantial differences in immune cell infiltration. Within the ATP2A3 cohort, B cells naive, T cells CD4 naive, plasma cells, T cells follicular helper, macrophages M0, mast cells activated exhibited significant differences between the NSOI and control groups (*p* < 0.05). However, T cells CD8, T cells CD4 memory resting, T cells CD4 memory activated, T cells regulatory (Tregs), T cells gamma–delta, natural killer (NK) cells resting, neutrophils, macrophages M1, dendritic cells resting, and dendritic cells activated did not show significant variance between the two groups (*p* > 0.05). Within the immune cell landscape, B cells naive, T cells CD4 naive, plasma cells, T cells follicular helper, macrophages M0, and mast cells activated were predominantly expressed in the treatment group. Conversely, B cells memory, NK cells activated, monocytes, macrophages M2, and mast cells resting were highly expressed in the control group (Figure 8a). Additionally, we constructed an immune infiltration correlation matrix and heatmap to further elucidate these relationships (Figure 8b,c). A Lollipop plot was utilized to illustrate the expression patterns of correlation coefficients, highlighting dendritic cells activated, NK cells resting, T cells follicular helper, T cells CD4 naive, B cells naive, and plasma cells (Figure 8d). In addition, we also performed correlation analysis of these model genes on immune cells (Figure 8e). B cells memory (*R* = −0.27, *p*=0.0059), macrophages M2 (*R* = − 0.49, *p*=1.9e − 07), mast cells resting (*R* = − 0.3, *p*=0.0023), monocytes (*R* = − 0.3, *p*=0.0021), and Tregs (*R* = −0.23, *p*=0.021) were positively associated with ATP2A3, whereas plasma cells (*R* = 0.5, *p*=1.3e − 07) and B cells naive ( = 0.27, *p*=0.0055) were negatively associated with ATP2A3 (Figure 8f; Table S7). To further elucidate the immunological implications of our findings, we expanded the discussion on the relationship between ATP2A3 expression and specific immune cell subsets, including Tregs, monocytes, and B cell subpopulations. ATP2A3 encodes a SERCA isoform, which regulates intracellular calcium homeostasis—a critical modulator of immune cell activation, differentiation, and function. Elevated expression of ATP2A3 may influence Treg stability and suppressive capacity via calcium-dependent signaling cascades such as NFAT and calcineurin pathways, which are essential for FOXP3 expression and Treg lineage commitment. In monocytes, ATP2A3-associated calcium dynamics may affect inflammatory cytokine production and antigen-presenting function, potentially shifting the balance between classical and nonclassical monocyte subsets. Similarly, alterations in calcium signaling mediated by ATP2A3 could modulate B cell maturation and antibody production, particularly impacting memory B cell formation through regulation of BCR signaling thresholds.

### 3.7. MR Analysis

**Figure 9:**
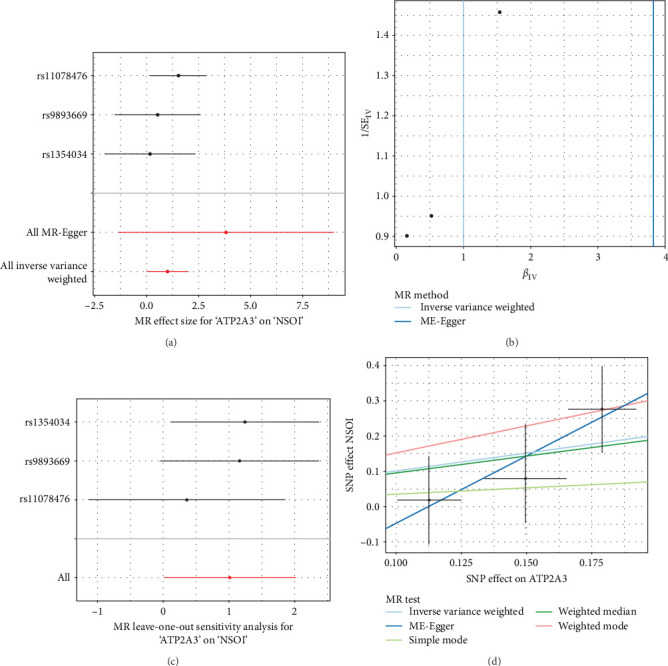
Mendelian randomization analysis. (a) Correlation rectangle plot. (b) Heatmap. (c) The expression patterns of correlation coefficient. (d) SNP effect on NSOI.

To address the potential influence of horizontal pleiotropy in the MR framework, we evaluated both the MR-Egger intercept and Cochran's *Q* statistic. The MR-Egger intercept test was performed to assess directional horizontal pleiotropy, with a nonsignificant intercept *p*-value indicating no evidence of unbalanced pleiotropic effects. In parallel, Cochran's *Q* statistic was computed to evaluate heterogeneity across IVs, which may reflect the presence of horizontal pleiotropy or violations of other IV assumptions. Together, these sensitivity analyses provided a robust assessment of potential pleiotropic bias, ensuring the validity of the causal inference. In examining the direct linkage between the ATP2A3 (*n* = 35,431) and NSOI (data were obtained from the eye and adnexa; *n* = 16,380,434) incidence, a forest plot was utilized for visual illustration, revealing a general symmetry in the data. Through sensitivity analysis employing the “leave-one-out” technique, it was determined that the omission of any individual SNP had a minimal effect on the results of the inverse variance-weighted (IVW) analysis, indicating that the remaining SNPs closely mirrored the overall dataset's findings. To further authenticate our outcomes, MR-Egger regression analysis was conducted, bolstering the integrity and reliability of our results and the chosen analytical framework (Figure 9a–d).

## 4. Discussions

NSOI, a a rare idiopathic inflammatory orbital disorder, is delineated by unilateral and symptomatic orbital edema, notably devoid of precedent viral or systemic perturbations. Critical exacerbations in this condition may be driven by optic nerve dysfunction. Despite its clinical pertinence, the molecular orchestrations governing this condition substantially remain an enigma [24]. An accumulating corpus of evidence posits that modulations in gene expression are pivotal in the pathogenesis of NSOI. ATP2A3, encoding the SERCA3, is essential for maintaining intracellular calcium homeostasis and mediating signal transduction. Calcium signaling governs a multitude of physiological processes and its dysregulation has been implicated in the pathogenesis of diverse disorders, including metabolic syndromes, neurodegenerative diseases, and various malignancies [25]. In the visual system—particularly within the retina, where calcium fluxes are tightly regulated—ATP2A3 plays a pivotal role in sustaining neuronal viability and functional integrity [26]. Disruptions in calcium homeostasis have been closely linked to the development of glaucoma, diabetic retinopathy, and AMD, all of which feature neuroinflammation, apoptotic cell death, and visual impairment [27]. Emerging evidence suggests that ATP2A3 is integral to retinal protection by modulating calcium dynamics in retinal pigment epithelial (RPE) cells and retinal ganglion cells. Impaired expression or function of ATP2A3 may exacerbate ER stress, thereby activating proapoptotic pathways that accelerate retinal degeneration [28]. In addition, ATP2A3 is suggested to influence intraocular pressure regulation, blood–retinal barrier integrity and optic nerve preservation via calcium-dependent signaling cascades [29]. These multifaceted roles underscore ATP2A3 as a crucial molecular node in the pathophysiology of ocular diseases. To further elucidate its role in orbital inflammatory pathology, we employed LASSO regression and WGCNA to identify disease-associated transcriptomic signatures in NSOI. Through rigorous cross-validation, 15 hub genes were identified, including ATP2A3, ASS1, CEBPA, CSTA, FAM46C, HSD11B1, IRX5, KCNN4, MMD, MPPED2, PHGDH, SFRP1, SIX1, TMEM30B, and WFDC2. Among these, ATP2A3 emerged as a particularly compelling candidate given its established involvement in calcium regulation, immune signaling, and inflammatory processes. The identification of ATP2A3 in the NSOI gene network not only reinforces its relevance in ocular immunopathology but also positions it as a promising target for diagnostic and therapeutic development. In conclusion, ATP2A3 acts as a critical modulator of calcium homeostasis and ER function in ocular tissues, bridging neurodegenerative, and inflammatory mechanisms. Its dysregulation contributes to the molecular pathogenesis of both retinal and orbital diseases, highlighting its potential as a biomarker and therapeutic target in immune-mediated ocular disorders.

In the complex landscape of NSOI, emerging evidence challenges conventional perspectives that primarily attribute heightened immune responses to CD4+ T cell activity. Instead, a more nuanced profile emerges, wherein the interplay of existing Tregs and imbalances in proinflammatory and regulatory factors, such as cytokines, predominates the immunological environment [30]. This dysregulated immune milieu may hinder effective immune reconstitution, increasing susceptibility to opportunistic infections, whether active, latent, or previously managed [31]. Conditions such as tuberculosis, cytomegalovirus (CMV) infections, progressive multifocal leukoencephalopathy, Kaposi's sarcoma, and various autoimmune disorders may exacerbate or elude detection, with CMV retinitis notably emerging as the most prevalent opportunistic infection linked to immunological recovery uveitis [32, 33]. Innovative therapies aimed at enhancing intracellular cyclic adenosine monophosphate (cAMP) levels present a promising strategy for alleviating chronic inflammation. Small-molecule phosphodiesterase-4 (PDE4) inhibitors, which prevent cAMP degradation, have shown efficacy across a spectrum of inflammatory diseases, including inflammatory bowel disease, atopic dermatitis, and rheumatoid arthritis [34, 35]. Building on our prior investigations, we meticulously analyzed the expression profile of ATP2A3 within the immunological microenvironment. A Lollipop plot was employed to visualize the expression patterns of correlation coefficients, revealing significant associations with activated dendritic cells, resting NK cells, follicular helper T cells, naive CD4+ T cells, naive B cells, and plasma cells (Figure 7d). Furthermore, we conducted correlation analyses of these model genes with immune cell types. Memory B cells, M2 macrophages, resting mast cells, monocytes, and Tregs exhibited positive correlations with ATP2A3, while plasma cells and naive B cells demonstrated negative associations. This intricate interplay between ATP2A3 and various immune cell types underscores the pivotal role of inflammation and immune responses in the pathophysiology of NSOI. Our findings illuminate potential pathways for targeted therapeutic interventions, emphasizing the critical importance of modulating the immune landscape in the development of effective treatment strategies for NSOI.

In embarking on an exploration of the under-researched interplay between biomarkers and NSOI, our study aims to chart a pioneering course in this rapidly expanding field. While the existing literature robustly employs bioinformatics to elucidate connections between immune responses and ocular diseases, a significant gap remains regarding the role of ATP2A3 in the context of NSOI [36–38]. Previous landmark studies have significantly advanced our understanding of the molecular architecture underlying NSOI. Liu et al. [36] leveraged WGCNA to identify hub genes implicated in NSOI pathophysiology, while Hu et al. [37] applied sophisticated computational pipelines to delineate 11 key genes associated with thyroid eye disease, a related orbital inflammatory condition. In parallel, Huang and Zhou [38] combined integrative bioinformatics with in vivo validation to uncover critical regulators such as CD44 and CDC42 in the context of diabetic retinopathy, underscoring the translational potential of network-based approaches in ocular disease research. Building upon these foundational contributions, our study offers a complementary and expanded perspective by identifying ATP2A3 as a novel immunometabolic regulator in NSOI. Unlike prior studies, we incorporate both network-based gene prioritization and MR—a methodological advance not previously employed in this context—to establish a putative causal relationship between ATP2A3 and NSOI susceptibility. Utilizing a robust transcriptomic dataset from the GEO, our analysis reveals consistent dysregulation of ATP2A3, suggesting its dual role in calcium homeostasis and immune modulation. This work not only reinforces the validity of gene network analysis in dissecting NSOI pathogenesis but also extends the field by proposing ATP2A3 as a candidate therapeutic target. Through methodological innovation and causal inference, we provide a refined molecular framework that complements and builds upon earlier studies, thereby contributing to a more nuanced understanding of the immune–metabolic interface in orbital inflammation. Amid the complex immunopathology of ocular inflammatory disorders, ATP2A3 has emerged as a compelling candidate in the pathogenesis of NSOI. As a critical regulator of intracellular calcium homeostasis with established roles in immune modulation, ATP2A3 may serve as a molecular bridge linking dysregulated immune responses to orbital tissue damage. By integrating transcriptomic profiling with predictive modeling, we reveal a significant differential expression of ATP2A3 in NSOI tissues compared to healthy controls, suggesting its potential utility as both a biomarker for disease stratification and a target for therapeutic intervention.

We acknowledge several limitations inherent to this study. First, our analyses relied primarily on publicly available transcriptomic datasets, which, despite rigorous normalization, remain vulnerable to batch effects and intersample variability. These technical artifacts may obscure subtle biological signals or introduce bias into differential expression analyses. Second, although our computational framework provides mechanistic insights into coexpression network, these findings lack experimental validation. The absence of confirmatory assays—such as quantitative PCR, western blotting, or immunohistochemistry—limits the interpretation of gene expression changes at both the transcript and protein levels. Furthermore, our analysis focused exclusively on protein-coding genes, thereby omitting the broader regulatory landscape governed by noncoding RNAs. Emerging evidence underscores the critical roles of long noncoding RNAs (lncRNAs) and microRNAs (miRNAs) in immune signaling, cellular metabolism, and inflammatory responses—dimensions that remain unexplored in the present work. Addressing these gaps will require future studies incorporating patient-derived tissues and in vitro functional assays to validate the immunometabolic roles of key gluconeogenesis-related genes. Parallel profiling of noncoding RNA species, alongside perturbation experiments using CRISPR/Cas9 or RNA interference, may further delineate the regulatory architecture underlying NSOI pathogenesis. In parallel, we sought to elucidate the role of ATP2A3 in ocular inflammation beyond the retinal context. We identified 15 hub genes with high network centrality in NSOI, among which ATP2A3 emerged as particularly notable. Given its established roles in calcium transport, immune modulation, and ER homeostasis, ATP2A3 may represent a mechanistic link between calcium dysregulation and immune-mediated tissue injury in the orbit. While these findings offer valuable theoretical insights, they also hold translational potential. The consistent dysregulation of ATP2A3 across a spectrum of ocular inflammatory and degenerative conditions suggests its utility as both a biomarker for disease stratification and a prospective therapeutic target. Modulation of ATP2A3 expression or activity could inform new strategies to attenuate ER stress, reestablish calcium equilibrium, and suppress neuroinflammation in ocular disease contexts.

## 5. Conclusions

In the context of NSOI, this study identifies ATP2A3 as a novel player in immune-mediated pathogenesis. We demonstrate that ATP2A3 is markedly dysregulated in NSOI tissues compared to normal controls, highlighting its prognostic significance within the inflammatory milieu.

## Data Availability

The datasets generated during and/or analyzed during the current study are available in the supporting information. The citation guidelines: www.kegg.jp/kegg/kegg1.html.

## Nomenclature

NSOI: Nonspecific orbital inflammation
GO: Gene Ontology
TCM: Traditional Chinese medicine
MF: Molecular functions
KEGG: Kyoto Encyclopedia of Genes and Genomes
GEO: Gene Expression Omnibus
ATP2A3: Sarcoplasmic/endoplasmic reticulum calcium ATPase 3
BP: Biological processes
CC: Cellular components
DEGs: Differentially expressed genes.

## Supporting Information

Supporting Information Appendix 1: The clinical characteristics of patients. Appendix 2: DEGs linked to NSOI. Appendix 3: InterGenes. Appendix 4: Analysis of enrichment. Appendix 5: Coexpression network. Appendix 6: GSEA analysis. Appendix 7: Immune Correlation Analysis.
