# Supplementary material for: Immune Regulation and ECM-Related Pathway Enrichment Reveal ATP2A3 as a Prognostic Biomarker for Nonspecific Orbital Inflammation: An Integrated Machine Learning and Mendelian Randomization Analysis
Source: Mediators Inflamm. 2025 Jun 13;2025:7061507. doi: 10.1155/mi/7061507 (PMC12181670; doi:10.1155/mi/7061507)
Supplement: Supporting Information — Appendix 1: The clinical characteristics of patients. Appendix 2: DEGs linked to NSOI. Appendix 3: InterGenes. Appendix 4: Analysis of enrichment. Appendix 5: Coexpression network. Appendix 6: GSEA analysis. Appendix 7: Immune Correlation Analysis. [file 7061507.f1.doc]

Integrated Multiple Machine Learning and Mendelian Randomization Reveal ATP2A3 as a Prognostic Biomarker for Nonspecific Orbital Inflammation

**Supplementary appendix to the manuscript**

Contents of supplementary appendix

[Appendix 1 3](#__RefHeading___Toc12687)

[The clinical characteristics of patients 3](#__RefHeading___Toc9860)

[Table S1. The clinical characteristics of patients. 3](#__RefHeading___Toc4740)

[Appendix 2 4](#__RefHeading___Toc30107)

[DEGs linked to NSOI 4](#__RefHeading___Toc5)

[Table S2. DEGs linked to NSOI. 4](#__RefHeading___Toc2792)

[Appendix 3 10](#__RefHeading___Toc23626)

[Table S3a.InterGenes. 10](#__RefHeading___Toc177)

[Appendix 4 11](#__RefHeading___Toc15816)

[Table S4a. Analysis of GO. 11](#__RefHeading___Toc27600)

[Table S4b. Analysis of KEGG. 29](#__RefHeading___Toc17726)

[Appendix 5 30](#__RefHeading___Toc5139)

[Table S5.Gene regulatory networks 30](#__RefHeading___Toc17702)

[Appendix 6 35](#__RefHeading___Toc3172)

[Table S6a. GO of GSEA analysis. 35](#__RefHeading___Toc19895)

[Table S6b. KEGG of GSEA analysis. 48](#__RefHeading___Toc4428)

[Appendix 7 51](#__RefHeading___Toc2514)

[Table S7. Immune Correlation Analysis. 51](#__RefHeading___Toc2204)

# Appendix 1

**The clinical characteristics of patients**

**Table S1.** **The clinical characteristics of patients.**

| GSE58331 | | GSE105149 | |
| --- | --- | --- | --- |
| Variables | Number of samples | Variables | Number of samples |
| Gender |  | Gender |  |
| Male/Female | 19/56 | Male/Female | 9/18 |
| Diagnosis |  | Diagnosis |  |
| NSOI/Normal | 75/29 | NSOI/Normal | 27/7 |
| Tissue |  | Tissue |  |
| Anterior Orbit/  Lacrimal gland | 33/42 | Anterior Orbit/  Lacrimal gland | 0/27 |

# Appendix 2

## **DEGs linked to NSOI**

**Table S2. DEGs linked to NSOI.**

| id | logFC | AveExpr | t | P.Value | adj.P.Val | B |
| --- | --- | --- | --- | --- | --- | --- |
| HLF | -1.610353935 | 4.514203368 | -11.31125638 | 1.73E-21 | 3.75E-17 | 38.10791255 |
| PGRMC2 | -1.312662555 | 4.193841849 | -10.26136165 | 8.89E-19 | 7.24E-15 | 32.05448142 |
| CYB5A | -1.016398535 | 4.902457831 | -10.24094873 | 1.00E-18 | 7.24E-15 | 31.93729473 |
| PGM1 | -1.343478593 | 5.618336972 | -10.09660583 | 2.35E-18 | 1.27E-14 | 31.10948695 |
| LARP6 | -0.960988227 | 4.294425693 | -9.890690894 | 7.92E-18 | 3.43E-14 | 29.93138602 |
| GULP1 | -0.883849252 | 3.367007863 | -9.853356856 | 9.87E-18 | 3.56E-14 | 29.7181804 |
| MGST1 | -1.759410241 | 4.920498638 | -9.77179663 | 1.59E-17 | 4.93E-14 | 29.25286501 |
| PEX19 | -1.023660372 | 5.236252528 | -9.747705491 | 1.84E-17 | 4.97E-14 | 29.11554443 |
| TUSC1 | -1.347177348 | 5.660676496 | -9.654470148 | 3.17E-17 | 7.63E-14 | 28.5846549 |
| C1orf198 | -1.123759298 | 6.38305599 | -9.456176831 | 1.01E-16 | 2.06E-13 | 27.45870877 |
| ADI1 | -0.684905035 | 5.299760613 | -9.430695872 | 1.17E-16 | 2.06E-13 | 27.31435555 |
| TGFBR3 | -1.818931005 | 5.39278535 | -9.429635103 | 1.18E-16 | 2.06E-13 | 27.30834786 |
| ADH1B | -2.087243769 | 4.297769655 | -9.421801457 | 1.24E-16 | 2.06E-13 | 27.26398611 |
| PRDX6 | -1.13444175 | 6.799394132 | -9.337709507 | 2.02E-16 | 3.12E-13 | 26.78825915 |
| SLC24A3 | -1.174661249 | 4.360708884 | -9.319775286 | 2.24E-16 | 3.24E-13 | 26.68691823 |
| EIF4EBP2 | -0.603792547 | 5.598891696 | -9.250917863 | 3.35E-16 | 4.53E-13 | 26.29822026 |
| CYSTM1 | -1.013519425 | 6.853163491 | -9.23023993 | 3.77E-16 | 4.68E-13 | 26.18161878 |
| NTRK2 | -1.227603896 | 4.604987935 | -9.220369693 | 3.99E-16 | 4.68E-13 | 26.12598188 |
| SSPN | -0.906042377 | 4.407599779 | -9.215591743 | 4.11E-16 | 4.68E-13 | 26.0990542 |
| RAB5B | -0.723075115 | 6.58963281 | -9.127992558 | 6.83E-16 | 7.39E-13 | 25.60593058 |
| C2orf40 | -2.020369435 | 4.739671919 | -9.118829269 | 7.20E-16 | 7.42E-13 | 25.55441111 |
| ANGPT1 | -0.893925881 | 3.307847816 | -9.110394923 | 7.56E-16 | 7.44E-13 | 25.50700089 |
| WLS | -1.179076987 | 5.23318877 | -9.082934572 | 8.86E-16 | 8.34E-13 | 25.35271639 |
| GSTM3 | -1.169210932 | 4.809026677 | -9.010371188 | 1.35E-15 | 1.22E-12 | 24.94556795 |
| RNF11 | -1.623643588 | 3.908796206 | -8.994408605 | 1.48E-15 | 1.26E-12 | 24.8561112 |
| SLC17A9 | 0.862749105 | 5.801984346 | 8.961289124 | 1.79E-15 | 1.44E-12 | 24.67063175 |
| RNASE4 | -1.570154648 | 5.339713254 | -8.934011637 | 2.09E-15 | 1.62E-12 | 24.51799987 |
| C2orf76 | -0.858031164 | 3.55539803 | -8.921303954 | 2.25E-15 | 1.68E-12 | 24.44693446 |
| FAXDC2 | -1.036158209 | 5.053485405 | -8.896623426 | 2.60E-15 | 1.87E-12 | 24.30898793 |
| THRB | -0.877072211 | 4.099635227 | -8.885823488 | 2.76E-15 | 1.92E-12 | 24.24865531 |
| APCDD1 | -1.517422375 | 5.806270179 | -8.881162301 | 2.84E-15 | 1.92E-12 | 24.22262205 |
| STXBP1 | -1.435828861 | 4.731128234 | -8.835649787 | 3.69E-15 | 2.42E-12 | 23.96861932 |
| ADH5 | -1.00515715 | 5.291857895 | -8.814425221 | 4.17E-15 | 2.65E-12 | 23.85028535 |
| ITGB5 | -0.880396918 | 4.746239512 | -8.776933203 | 5.17E-15 | 3.20E-12 | 23.64144324 |
| CAV2 | -1.386816596 | 4.828781633 | -8.761486467 | 5.65E-15 | 3.34E-12 | 23.55547093 |
| NGFRAP1 | -1.267418152 | 6.74946274 | -8.759707738 | 5.70E-15 | 3.34E-12 | 23.54557368 |
| TIMP4 | -1.880645299 | 3.846524614 | -8.735148502 | 6.57E-15 | 3.74E-12 | 23.40897746 |
| ENPP6 | -1.330917992 | 2.75974725 | -8.721928886 | 7.08E-15 | 3.93E-12 | 23.33549533 |
| NPR3 | -1.678804175 | 3.530898307 | -8.690571199 | 8.48E-15 | 4.59E-12 | 23.16131648 |
| FAM213A | -1.129763179 | 5.364323531 | -8.677680776 | 9.13E-15 | 4.82E-12 | 23.08976701 |
| NECAB1 | -1.098895958 | 2.184179631 | -8.641126523 | 1.12E-14 | 5.66E-12 | 22.88703439 |
| TSKU | -1.469259022 | 5.680635735 | -8.622995657 | 1.25E-14 | 6.04E-12 | 22.78657076 |
| SIK2 | -0.701924476 | 4.408789635 | -8.621961435 | 1.25E-14 | 6.04E-12 | 22.78084195 |
| DEF6 | 0.974197777 | 6.723024224 | 8.615072644 | 1.30E-14 | 6.14E-12 | 22.74268836 |
| HADH | -1.059785769 | 4.987685528 | -8.605090306 | 1.38E-14 | 6.36E-12 | 22.68741693 |
| GHR | -2.085336976 | 3.679512872 | -8.598071676 | 1.44E-14 | 6.49E-12 | 22.64856655 |
| CAB39L | -0.970409181 | 5.027229943 | -8.588128562 | 1.52E-14 | 6.73E-12 | 22.59354412 |
| OLFML2A | -1.447956663 | 5.374092376 | -8.532691327 | 2.09E-14 | 9.04E-12 | 22.28711594 |
| ALDH2 | -1.511307293 | 7.134622391 | -8.522234849 | 2.22E-14 | 9.41E-12 | 22.22938451 |
| GPAM | -2.029674594 | 4.096641238 | -8.503352887 | 2.47E-14 | 1.01E-11 | 22.12518916 |
| PPAP2B | -1.675900129 | 6.73550852 | -8.498218543 | 2.54E-14 | 1.01E-11 | 22.09686869 |
| PNPLA4 | -0.59252103 | 4.682154959 | -8.496659287 | 2.56E-14 | 1.01E-11 | 22.08826905 |
| MXRA7 | -0.924816576 | 5.057643453 | -8.496333383 | 2.57E-14 | 1.01E-11 | 22.08647167 |
| TNMD | -1.170932848 | 2.79412065 | -8.477526313 | 2.86E-14 | 1.10E-11 | 21.98278555 |
| FAM127B | -0.836330745 | 5.455579826 | -8.445954272 | 3.42E-14 | 1.26E-11 | 21.80888303 |
| ATXN1L | -1.148932844 | 5.382482793 | -8.445596266 | 3.42E-14 | 1.26E-11 | 21.80691224 |
| CACHD1 | -0.918633179 | 4.351842576 | -8.444916698 | 3.44E-14 | 1.26E-11 | 21.80317135 |
| RABL3 | -0.76477261 | 4.217499744 | -8.437423655 | 3.59E-14 | 1.29E-11 | 21.76192982 |
| BNIP3L | -1.171017334 | 6.83058701 | -8.432855178 | 3.68E-14 | 1.31E-11 | 21.73679061 |
| PPP1R1A | -2.092068302 | 5.874807824 | -8.426871989 | 3.81E-14 | 1.31E-11 | 21.703873 |
| CDR1 | -2.007220103 | 6.372376505 | -8.424111633 | 3.87E-14 | 1.31E-11 | 21.68868886 |
| LIMD2 | 1.097852248 | 7.544212571 | 8.392520443 | 4.63E-14 | 1.51E-11 | 21.51502339 |
| LINC01133 | -0.796378691 | 3.322647183 | -8.391151773 | 4.66E-14 | 1.51E-11 | 21.50750407 |
| SNRNP70 | 0.70068956 | 7.708397451 | 8.380517655 | 4.95E-14 | 1.58E-11 | 21.44909469 |
| TCIRG1 | 0.874414665 | 6.859804531 | 8.374730055 | 5.12E-14 | 1.61E-11 | 21.41731535 |
| CPE | -1.319670868 | 4.765008159 | -8.370830041 | 5.23E-14 | 1.62E-11 | 21.39590456 |
| IGFBP6 | -1.961099426 | 6.818834642 | -8.359696328 | 5.57E-14 | 1.68E-11 | 21.33479876 |
| ALDH3A2 | -0.846722995 | 5.661131212 | -8.359375145 | 5.58E-14 | 1.68E-11 | 21.33303638 |
| HEBP2 | -0.737064398 | 5.65704944 | -8.355472418 | 5.71E-14 | 1.69E-11 | 21.31162323 |
| IGLL3P | 1.56983869 | 9.04712142 | 8.352749199 | 5.79E-14 | 1.70E-11 | 21.29668359 |
| PBX1 | -1.343818174 | 6.707438158 | -8.338093375 | 6.29E-14 | 1.79E-11 | 21.21630828 |
| ADIPOR2 | -0.779643941 | 5.579097688 | -8.332421419 | 6.50E-14 | 1.83E-11 | 21.18521438 |
| LEPR | -2.196457234 | 4.100626463 | -8.320159453 | 6.97E-14 | 1.93E-11 | 21.1180171 |
| PLIN1 | -3.428900663 | 6.527600865 | -8.31587091 | 7.14E-14 | 1.96E-11 | 21.09452285 |
| PHYH | -1.376920113 | 4.544949985 | -8.313167669 | 7.25E-14 | 1.96E-11 | 21.07971549 |
| THSD4 | -0.907685591 | 3.741077646 | -8.306771715 | 7.51E-14 | 2.01E-11 | 21.04468704 |
| ZBP1 | 0.999159511 | 5.954012704 | 8.303042954 | 7.67E-14 | 2.03E-11 | 21.02426994 |
| TMTC1 | -0.830072017 | 4.09551352 | -8.293273725 | 8.11E-14 | 2.12E-11 | 20.97079199 |
| TTC28 | -0.733356407 | 4.741271535 | -8.285852353 | 8.45E-14 | 2.18E-11 | 20.93018024 |
| FTO | -1.019943309 | 7.223424592 | -8.267442673 | 9.38E-14 | 2.39E-11 | 20.82948901 |
| TMEM100 | -1.308636492 | 2.69378546 | -8.256935276 | 9.95E-14 | 2.46E-11 | 20.77205217 |
| PCOLCE2 | -1.660255441 | 3.08235546 | -8.248102431 | 1.05E-13 | 2.54E-11 | 20.72378766 |
| FAM149A | -0.687057341 | 4.081070628 | -8.239954554 | 1.10E-13 | 2.63E-11 | 20.67928116 |
| TRHDE-AS1 | -1.600575076 | 3.139981534 | -8.230201339 | 1.16E-13 | 2.75E-11 | 20.62602499 |
| UGDH | -1.114179369 | 4.167768621 | -8.207172942 | 1.32E-13 | 3.10E-11 | 20.5003651 |
| MEST | -1.997677126 | 4.12449204 | -8.203746443 | 1.34E-13 | 3.13E-11 | 20.4816777 |
| PLSCR4 | -1.322633928 | 3.027550471 | -8.200132343 | 1.37E-13 | 3.16E-11 | 20.46197001 |
| LOC284825 | -1.423316741 | 3.128099914 | -8.194540807 | 1.41E-13 | 3.22E-11 | 20.43148512 |
| FAM162A | -0.602165048 | 4.676084678 | -8.180849768 | 1.53E-13 | 3.41E-11 | 20.35687167 |
| TFDP2 | -0.654772711 | 5.330022448 | -8.175822392 | 1.57E-13 | 3.47E-11 | 20.32948407 |
| CETN2 | -1.111002174 | 5.764694847 | -8.166936194 | 1.65E-13 | 3.61E-11 | 20.28108881 |
| CHRDL1 | -1.802913855 | 5.881081887 | -8.153134094 | 1.78E-13 | 3.86E-11 | 20.20595655 |
| FKBP1B | -0.988609372 | 3.169299392 | -8.122950202 | 2.11E-13 | 4.53E-11 | 20.04180132 |
| SC5D | -0.957747145 | 4.862732059 | -8.11054878 | 2.27E-13 | 4.74E-11 | 19.97441703 |
| GBE1 | -1.206515335 | 3.138216123 | -8.109365647 | 2.28E-13 | 4.74E-11 | 19.96799023 |
| NAP1L5 | -0.830324142 | 3.401922156 | -8.108105012 | 2.30E-13 | 4.74E-11 | 19.9611428 |
| CORO2B | -1.14974774 | 4.457670625 | -8.084250414 | 2.62E-13 | 5.36E-11 | 19.83164083 |
| HNMT | -0.745349138 | 4.137457412 | -8.076268991 | 2.74E-13 | 5.55E-11 | 19.788341 |
| SNX21 | -0.845902167 | 4.291105067 | -8.069006405 | 2.86E-13 | 5.73E-11 | 19.74895396 |
| ADIPOQ | -3.146730234 | 4.178438995 | -8.056287349 | 3.07E-13 | 6.10E-11 | 19.68000498 |
| ACAP1 | 0.998561996 | 6.566564418 | 8.046983899 | 3.23E-13 | 6.31E-11 | 19.62959603 |
| NDN | -0.87724491 | 5.663765846 | -8.038455573 | 3.39E-13 | 6.53E-11 | 19.58340506 |
| ABL1 | -0.830955193 | 7.623280161 | -8.029940922 | 3.56E-13 | 6.75E-11 | 19.5373055 |
| TRAM2-AS1 | -0.605362555 | 3.813665792 | -8.027718052 | 3.60E-13 | 6.78E-11 | 19.52527341 |
| TMEM64 | -0.687815273 | 3.857818067 | -8.020379304 | 3.75E-13 | 7.00E-11 | 19.48555823 |
| IGHM | 2.063147489 | 8.386564803 | 8.003492705 | 4.12E-13 | 7.63E-11 | 19.39422216 |
| SPATA6 | -0.618212493 | 4.085956726 | -7.995471622 | 4.31E-13 | 7.91E-11 | 19.35086198 |
| PPAP2A | -1.204010759 | 5.928522518 | -7.985877414 | 4.55E-13 | 8.26E-11 | 19.29901839 |
| KLB | -1.206837758 | 2.3980831 | -7.984713361 | 4.58E-13 | 8.26E-11 | 19.2927298 |
| ALDH6A1 | -0.851954294 | 4.809909527 | -7.98238054 | 4.64E-13 | 8.30E-11 | 19.28012814 |
| HPGDS | -1.070710641 | 4.211652662 | -7.967355009 | 5.04E-13 | 8.95E-11 | 19.1989937 |
| SEMA3G | -1.349625448 | 6.0151368 | -7.930739799 | 6.18E-13 | 1.09E-10 | 19.00151293 |
| IGSF10 | -1.014959765 | 4.246712545 | -7.924409291 | 6.40E-13 | 1.12E-10 | 18.96740367 |
| ALDH1A1 | -1.258644621 | 5.573065432 | -7.919786946 | 6.57E-13 | 1.13E-10 | 18.94250444 |
| ACSS3 | -0.806071773 | 3.269352631 | -7.919541103 | 6.58E-13 | 1.13E-10 | 18.94118031 |
| F8 | -1.141593057 | 4.052017986 | -7.913958701 | 6.78E-13 | 1.16E-10 | 18.91111701 |
| PPL | -1.601374526 | 6.562138156 | -7.905564555 | 7.11E-13 | 1.19E-10 | 18.86592616 |
| TMEM255A | -1.046824303 | 2.004402175 | -7.891439298 | 7.69E-13 | 1.27E-10 | 18.78992126 |
| ZNF704 | -0.794849336 | 4.385102998 | -7.878443759 | 8.26E-13 | 1.36E-10 | 18.72003971 |
| NDNF | -1.849819133 | 4.477983177 | -7.876937998 | 8.33E-13 | 1.36E-10 | 18.71194548 |
| POLR2K | -0.760433266 | 5.322701202 | -7.866226967 | 8.84E-13 | 1.43E-10 | 18.65438492 |
| NFIA | -0.918717199 | 5.07263018 | -7.846254768 | 9.88E-13 | 1.58E-10 | 18.54713364 |
| AKAP12 | -0.921307622 | 3.845745533 | -7.845280064 | 9.93E-13 | 1.58E-10 | 18.54190207 |
| GPR146 | -0.880119377 | 5.980325199 | -7.844086037 | 1.00E-12 | 1.58E-10 | 18.53549366 |
| AMOTL2 | -1.321763723 | 5.358988685 | -7.83398014 | 1.06E-12 | 1.66E-10 | 18.48126943 |
| GUSBP11 | 0.616316257 | 6.444203048 | 7.812114599 | 1.19E-12 | 1.86E-10 | 18.36403815 |
| ARHGAP21 | -0.89262672 | 5.425183505 | -7.791563924 | 1.34E-12 | 2.07E-10 | 18.25397007 |
| FMO3 | -0.948492777 | 3.080361066 | -7.781458068 | 1.41E-12 | 2.16E-10 | 18.19988441 |
| PCDH18 | -1.338339975 | 5.513538487 | -7.746978904 | 1.71E-12 | 2.55E-10 | 18.0155582 |
| SH3D19 | -1.221612829 | 5.830703648 | -7.729296916 | 1.88E-12 | 2.79E-10 | 17.9211529 |
| SRI | -0.683848646 | 4.996707995 | -7.727173537 | 1.91E-12 | 2.79E-10 | 17.90982168 |
| ZAP70 | 0.953099302 | 5.505380549 | 7.726926481 | 1.91E-12 | 2.79E-10 | 17.90850337 |
| MAMDC2 | -1.783453576 | 3.226921941 | -7.723171235 | 1.95E-12 | 2.83E-10 | 17.88846705 |
| C11orf74 | -0.729413069 | 2.811820315 | -7.719806518 | 1.99E-12 | 2.87E-10 | 17.87051766 |
| TCF7L1 | -1.198011086 | 6.199290779 | -7.710686409 | 2.09E-12 | 2.97E-10 | 17.82188096 |
| OLFML1 | -1.485744359 | 3.40996427 | -7.707226486 | 2.13E-12 | 2.98E-10 | 17.8034354 |
| MICAL1 | 0.83533227 | 6.354860607 | 7.700680161 | 2.21E-12 | 3.04E-10 | 17.76854446 |
| MTURN | -1.052707067 | 5.520481198 | -7.700346494 | 2.21E-12 | 3.04E-10 | 17.76676638 |
| PRICKLE2 | -0.696541617 | 4.069836711 | -7.699949325 | 2.21E-12 | 3.04E-10 | 17.76464993 |
| MN1 | -1.694489336 | 5.335466566 | -7.693278366 | 2.30E-12 | 3.13E-10 | 17.72910802 |
| ACSL1 | -1.477090472 | 6.824143202 | -7.689108639 | 2.35E-12 | 3.18E-10 | 17.70689847 |
| VKORC1L1 | -0.924491551 | 5.098877052 | -7.68236329 | 2.44E-12 | 3.28E-10 | 17.67098022 |
| S100B | -0.854353294 | 3.162203351 | -7.679736229 | 2.47E-12 | 3.30E-10 | 17.65699477 |
| PARVA | -0.811148874 | 5.173577744 | -7.678838489 | 2.49E-12 | 3.30E-10 | 17.65221599 |
| GPC6 | -0.9672403 | 4.464904831 | -7.668934071 | 2.63E-12 | 3.47E-10 | 17.59950816 |
| RUNX1-IT1 | 1.067424483 | 5.034065395 | 7.659271408 | 2.77E-12 | 3.63E-10 | 17.54811284 |
| MZB1 | 1.184012526 | 7.156046818 | 7.656902794 | 2.81E-12 | 3.65E-10 | 17.53551821 |
| OMD | -1.441537337 | 3.963588854 | -7.655376586 | 2.83E-12 | 3.65E-10 | 17.52740372 |
| ACACB | -1.145744604 | 6.180892504 | -7.655256118 | 2.83E-12 | 3.65E-10 | 17.52676324 |
| CIDEC | -2.518032405 | 6.081025216 | -7.63695015 | 3.13E-12 | 3.98E-10 | 17.42948555 |
| ZFYVE21 | -0.877304669 | 5.679397169 | -7.635995417 | 3.15E-12 | 3.98E-10 | 17.42441466 |
| TUBB2A | -1.716435519 | 4.469355074 | -7.625374385 | 3.34E-12 | 4.17E-10 | 17.36802011 |
| SEPT10 | -0.992951519 | 4.443727107 | -7.613586009 | 3.56E-12 | 4.43E-10 | 17.30546417 |
| FAM127A | -0.981866353 | 7.359206684 | -7.606589304 | 3.70E-12 | 4.57E-10 | 17.26835403 |
| EFHD1 | -1.226578732 | 6.427935118 | -7.601088909 | 3.81E-12 | 4.64E-10 | 17.23918988 |
| LRRN4CL | -1.818547865 | 4.563236942 | -7.599890567 | 3.83E-12 | 4.64E-10 | 17.23283717 |
| LGALSL | -0.806664209 | 5.096729085 | -7.599589254 | 3.84E-12 | 4.64E-10 | 17.2312399 |
| USP53 | -0.926040448 | 3.567917957 | -7.599005044 | 3.85E-12 | 4.64E-10 | 17.22814305 |
| FABP4 | -2.31878955 | 5.448320028 | -7.597449012 | 3.89E-12 | 4.65E-10 | 17.21989513 |
| EPHX1 | -1.22861661 | 7.527997085 | -7.589160072 | 4.07E-12 | 4.84E-10 | 17.1759702 |
| BC022047 | -1.274230551 | 4.838952691 | -7.585855202 | 4.14E-12 | 4.90E-10 | 17.15846237 |
| CFH | -1.527688256 | 5.542662171 | -7.576171715 | 4.37E-12 | 5.11E-10 | 17.1071811 |
| C4orf3 | -0.82743221 | 6.381095868 | -7.575351835 | 4.39E-12 | 5.11E-10 | 17.10284045 |
| YAP1 | -1.079735399 | 5.740510438 | -7.57520891 | 4.39E-12 | 5.11E-10 | 17.10208379 |
| IRF3 | 0.64607348 | 7.019733028 | 7.57077813 | 4.50E-12 | 5.19E-10 | 17.0786296 |
| MYO1G | 0.822584161 | 5.163149919 | 7.564079572 | 4.66E-12 | 5.34E-10 | 17.04318162 |
| AR | -1.026885183 | 4.036505341 | -7.558216312 | 4.82E-12 | 5.49E-10 | 17.01216443 |
| MAOA | -1.790504794 | 5.676085138 | -7.552255353 | 4.98E-12 | 5.64E-10 | 16.98064049 |
| BMPER | -0.837463908 | 2.662599035 | -7.546064417 | 5.15E-12 | 5.80E-10 | 16.94791111 |
| RLTPR | 0.820772191 | 5.988639923 | 7.540658691 | 5.30E-12 | 5.89E-10 | 16.91934187 |
| SVIL | -0.804133925 | 5.424383278 | -7.538583277 | 5.36E-12 | 5.92E-10 | 16.90837555 |
| EAPP | -0.988745253 | 5.684455163 | -7.529433637 | 5.64E-12 | 6.16E-10 | 16.86004437 |
| C1RL-AS1 | 0.643439222 | 4.778763387 | 7.519070951 | 5.96E-12 | 6.49E-10 | 16.80533471 |
| GYG2 | -1.341313088 | 4.670950769 | -7.517208997 | 6.02E-12 | 6.52E-10 | 16.79550784 |
| THRSP | -1.699990811 | 4.754455709 | -7.515509827 | 6.08E-12 | 6.55E-10 | 16.78654098 |
| TMEM30B | -0.794657165 | 5.44560466 | -7.505557501 | 6.42E-12 | 6.88E-10 | 16.73403741 |
| LINC00094 | -0.934555102 | 5.048270742 | -7.496439089 | 6.75E-12 | 7.19E-10 | 16.68595847 |
| KLF11 | -0.676194192 | 4.765215163 | -7.493840911 | 6.84E-12 | 7.23E-10 | 16.67226341 |
| UBTD2 | -0.74594916 | 4.471515126 | -7.493632468 | 6.85E-12 | 7.23E-10 | 16.67116479 |
| GNG12 | -0.796505318 | 5.977533342 | -7.491732452 | 6.92E-12 | 7.25E-10 | 16.66115113 |
| FAM13A | -0.777788383 | 4.34676589 | -7.491558443 | 6.93E-12 | 7.25E-10 | 16.6602341 |
| CYB5D1 | -0.652292201 | 4.60645814 | -7.485075367 | 7.18E-12 | 7.47E-10 | 16.62607462 |
| C15orf61 | -1.016366785 | 4.350050873 | -7.470609185 | 7.76E-12 | 8.00E-10 | 16.54989642 |
| AQPEP | -1.204041285 | 2.523898944 | -7.469691169 | 7.80E-12 | 8.01E-10 | 16.54506428 |
| TMC6 | 0.857032245 | 6.224018513 | 7.4636286 | 8.06E-12 | 8.20E-10 | 16.51315906 |
| TBC1D10C | 1.483432316 | 6.538256751 | 7.447947899 | 8.78E-12 | 8.88E-10 | 16.43068733 |
| ALAD | -0.66084176 | 5.563993129 | -7.447066133 | 8.82E-12 | 8.89E-10 | 16.4260519 |
| COPRS | -0.668754777 | 5.448120166 | -7.442441136 | 9.05E-12 | 9.07E-10 | 16.40174215 |
| ITGB2-AS1 | 1.064661616 | 5.749926178 | 7.440845226 | 9.12E-12 | 9.11E-10 | 16.39335526 |
| ADAMTS5 | -1.216974916 | 2.99798882 | -7.436071876 | 9.36E-12 | 9.24E-10 | 16.36827468 |
| ANO6 | -0.980592845 | 4.60156479 | -7.435596816 | 9.39E-12 | 9.24E-10 | 16.36577895 |
| SMARCA1 | -0.691066958 | 3.27514294 | -7.433444384 | 9.50E-12 | 9.31E-10 | 16.35447197 |
| SYNC | -0.98556979 | 3.265990047 | -7.423286879 | 1.00E-11 | 9.79E-10 | 16.30113208 |
| TMEM14A | -0.78254482 | 3.771301351 | -7.421019277 | 1.02E-11 | 9.87E-10 | 16.28922849 |
| HSDL2 | -0.712078881 | 3.06864213 | -7.418192234 | 1.03E-11 | 9.97E-10 | 16.27439032 |
| EPB41L1 | -0.912141033 | 5.525276985 | -7.416950129 | 1.04E-11 | 1.00E-09 | 16.26787169 |
| PLAT | -1.715753062 | 4.722004862 | -7.412716865 | 1.06E-11 | 1.02E-09 | 16.24565882 |
| PID1 | -0.618303195 | 3.582911196 | -7.393767239 | 1.18E-11 | 1.12E-09 | 16.14629208 |
| PER3 | -0.726267546 | 4.298989431 | -7.392355762 | 1.19E-11 | 1.12E-09 | 16.13889501 |
| ARL2 | -0.703341019 | 6.328549716 | -7.38311628 | 1.25E-11 | 1.17E-09 | 16.09048898 |
| RPS27L | -0.923441945 | 4.564603762 | -7.378245792 | 1.28E-11 | 1.20E-09 | 16.06498272 |
| ITGAX | 0.601547259 | 4.614357493 | 7.376589784 | 1.29E-11 | 1.21E-09 | 16.05631201 |
| FHL1 | -1.978402712 | 6.281394618 | -7.375362121 | 1.30E-11 | 1.21E-09 | 16.04988462 |
| PCYOX1 | -1.165948451 | 5.539007334 | -7.370763714 | 1.33E-11 | 1.23E-09 | 16.02581388 |
| RBP4 | -1.233372469 | 4.485850701 | -7.363766987 | 1.39E-11 | 1.28E-09 | 15.98920132 |
| SPIB | 0.909273226 | 4.255467612 | 7.360074539 | 1.41E-11 | 1.30E-09 | 15.96988547 |
| MIAT | 0.941484878 | 5.14076145 | 7.35428898 | 1.46E-11 | 1.33E-09 | 15.93962857 |
| LRCH4 | 0.594556023 | 7.004234027 | 7.348357244 | 1.51E-11 | 1.37E-09 | 15.90861786 |
| NEK7 | -0.990213116 | 3.471725902 | -7.339232961 | 1.58E-11 | 1.43E-09 | 15.86093786 |
| LYVE1 | -2.142445315 | 5.49862036 | -7.336193392 | 1.61E-11 | 1.44E-09 | 15.84505993 |
| CAT | -0.933833128 | 5.305581803 | -7.332899327 | 1.64E-11 | 1.46E-09 | 15.8278558 |
| TMEM132C | -1.320233525 | 4.133549582 | -7.332803511 | 1.64E-11 | 1.46E-09 | 15.82735543 |
| APOOL | -0.74981666 | 4.188843761 | -7.303747443 | 1.92E-11 | 1.67E-09 | 15.67574888 |
| SORBS1 | -1.072337929 | 5.646683103 | -7.302677643 | 1.93E-11 | 1.67E-09 | 15.67017197 |
| EID1 | -0.934694252 | 6.064050137 | -7.300336251 | 1.95E-11 | 1.68E-09 | 15.65796745 |
| STS | -0.634855584 | 4.500728269 | -7.300181523 | 1.95E-11 | 1.68E-09 | 15.65716099 |
| DIAPH2 | -0.939765522 | 5.169867595 | -7.29082236 | 2.05E-11 | 1.76E-09 | 15.60839386 |

# Appendix 3

**LASSO and** **SVM-RFE genes**

**Table S3a.InterGenes.**

| TMEM30B | MPPED2 | ATP2A3 | IRX5 | HSD11B1 |
| --- | --- | --- | --- | --- |
| SIX1 | CSTA | WFDC2 | FAM46C | KCNN4 |
| MMD | CEBPA | ASS1 | PHGDH | SFRP1 |

# Appendix 4

**Analysis of enrichment**

**Table S4a. Analysis of GO.**

| ONTOLOGY | ID | Description | BgRatio | pvalue | qvalue |
| --- | --- | --- | --- | --- | --- |
| BP | GO:0060541 | respiratory system development | 211/18614 | 1.06E-05 | 0.002324429 |
| BP | GO:0045600 | positive regulation of fat cell differentiation | 71/18614 | 1.48E-05 | 0.002324429 |
| BP | GO:0000050 | urea cycle | 10/18614 | 2.02E-05 | 0.002324429 |
| BP | GO:0019627 | urea metabolic process | 12/18614 | 2.96E-05 | 0.002324429 |
| BP | GO:0071941 | nitrogen cycle metabolic process | 12/18614 | 2.96E-05 | 0.002324429 |
| BP | GO:0045598 | regulation of fat cell differentiation | 146/18614 | 0.000127626 | 0.008351678 |
| BP | GO:0003401 | axis elongation | 28/18614 | 0.000168465 | 0.009202703 |
| BP | GO:0048732 | gland development | 441/18614 | 0.000187508 | 0.009202703 |
| BP | GO:0045664 | regulation of neuron differentiation | 194/18614 | 0.000295184 | 0.012877601 |
| BP | GO:0048701 | embryonic cranial skeleton morphogenesis | 45/18614 | 0.000438274 | 0.014045181 |
| BP | GO:0009066 | aspartate family amino acid metabolic process | 47/18614 | 0.000478183 | 0.014045181 |
| BP | GO:0048705 | skeletal system morphogenesis | 229/18614 | 0.000479823 | 0.014045181 |
| BP | GO:0071356 | cellular response to tumor necrosis factor | 235/18614 | 0.000517453 | 0.014045181 |
| BP | GO:0060688 | regulation of morphogenesis of a branching structure | 51/18614 | 0.000563111 | 0.014045181 |
| BP | GO:0045332 | phospholipid translocation | 52/18614 | 0.000585405 | 0.014045181 |
| BP | GO:0045444 | fat cell differentiation | 246/18614 | 0.000591275 | 0.014045181 |
| BP | GO:0050873 | brown fat cell differentiation | 53/18614 | 0.000608122 | 0.014045181 |
| BP | GO:0034612 | response to tumor necrosis factor | 257/18614 | 0.000671551 | 0.014531889 |
| BP | GO:0034204 | lipid translocation | 57/18614 | 0.000703219 | 0.014531889 |
| BP | GO:0097035 | regulation of membrane lipid distribution | 62/18614 | 0.00083156 | 0.016324843 |
| BP | GO:1905330 | regulation of morphogenesis of an epithelium | 65/18614 | 0.000913592 | 0.017081199 |
| BP | GO:1901607 | alpha-amino acid biosynthetic process | 70/18614 | 0.001058647 | 0.018893549 |
| BP | GO:1904888 | cranial skeletal system development | 72/18614 | 0.001119574 | 0.018910044 |
| BP | GO:0001822 | kidney development | 310/18614 | 0.001155895 | 0.018910044 |
| BP | GO:0072001 | renal system development | 320/18614 | 0.00126665 | 0.019348076 |
| BP | GO:0008652 | amino acid biosynthetic process | 78/18614 | 0.001312263 | 0.019348076 |
| BP | GO:0009064 | glutamine family amino acid metabolic process | 79/18614 | 0.001345818 | 0.019348076 |
| BP | GO:0071230 | cellular response to amino acid stimulus | 80/18614 | 0.001379782 | 0.019348076 |
| BP | GO:0061053 | somite development | 86/18614 | 0.001592153 | 0.02155619 |
| BP | GO:0071229 | cellular response to acid chemical | 89/18614 | 0.001703833 | 0.022299288 |
| BP | GO:0014033 | neural crest cell differentiation | 95/18614 | 0.001938116 | 0.023116938 |
| BP | GO:0048704 | embryonic skeletal system morphogenesis | 95/18614 | 0.001938116 | 0.023116938 |
| BP | GO:0001657 | ureteric bud development | 97/18614 | 0.002019432 | 0.023116938 |
| BP | GO:0072163 | mesonephric epithelium development | 98/18614 | 0.002060692 | 0.023116938 |
| BP | GO:0072164 | mesonephric tubule development | 98/18614 | 0.002060692 | 0.023116938 |
| BP | GO:0001823 | mesonephros development | 102/18614 | 0.002229733 | 0.024318435 |
| BP | GO:0015914 | phospholipid transport | 104/18614 | 0.002316647 | 0.024583484 |
| BP | GO:1901655 | cellular response to ketone | 107/18614 | 0.00245 | 0.025314403 |
| BP | GO:1903707 | negative regulation of hemopoiesis | 115/18614 | 0.002823003 | 0.027978557 |
| BP | GO:0052547 | regulation of peptidase activity | 425/18614 | 0.002850362 | 0.027978557 |
| BP | GO:0032355 | response to estradiol | 120/18614 | 0.003068895 | 0.028689169 |
| BP | GO:0043200 | response to amino acid | 120/18614 | 0.003068895 | 0.028689169 |
| BP | GO:0071901 | negative regulation of protein serine/threonine kinase activity | 123/18614 | 0.003221113 | 0.029411872 |
| BP | GO:0001838 | embryonic epithelial tube formation | 126/18614 | 0.003376826 | 0.029539444 |
| BP | GO:0048568 | embryonic organ development | 453/18614 | 0.003414164 | 0.029539444 |
| BP | GO:0048706 | embryonic skeletal system development | 129/18614 | 0.003536022 | 0.029539444 |
| BP | GO:2000027 | regulation of animal organ morphogenesis | 129/18614 | 0.003536022 | 0.029539444 |
| BP | GO:0048565 | digestive tract development | 134/18614 | 0.003809053 | 0.031157385 |
| BP | GO:0001101 | response to acid chemical | 136/18614 | 0.003920951 | 0.031418145 |
| BP | GO:0072175 | epithelial tube formation | 138/18614 | 0.004034377 | 0.031680479 |
| BP | GO:0001889 | liver development | 142/18614 | 0.004265801 | 0.032578518 |
| BP | GO:0061008 | hepaticobiliary system development | 145/18614 | 0.004443354 | 0.032578518 |
| BP | GO:0055123 | digestive system development | 146/18614 | 0.004503295 | 0.032578518 |
| BP | GO:0072073 | kidney epithelium development | 146/18614 | 0.004503295 | 0.032578518 |
| BP | GO:0045667 | regulation of osteoblast differentiation | 147/18614 | 0.004563613 | 0.032578518 |
| BP | GO:0015748 | organophosphate ester transport | 149/18614 | 0.004685379 | 0.032850498 |
| BP | GO:0016331 | morphogenesis of embryonic epithelium | 152/18614 | 0.004870848 | 0.032891189 |
| BP | GO:0035148 | tube formation | 154/18614 | 0.004996368 | 0.032891189 |
| BP | GO:0007584 | response to nutrient | 156/18614 | 0.005123382 | 0.032891189 |
| BP | GO:0021915 | neural tube development | 163/18614 | 0.005579649 | 0.032891189 |
| BP | GO:0060538 | skeletal muscle organ development | 178/18614 | 0.006618118 | 0.032891189 |
| BP | GO:0007219 | Notch signaling pathway | 179/18614 | 0.006690266 | 0.032891189 |
| BP | GO:0021561 | facial nerve development | 10/18614 | 0.006963761 | 0.032891189 |
| BP | GO:0021610 | facial nerve morphogenesis | 10/18614 | 0.006963761 | 0.032891189 |
| BP | GO:0043587 | tongue morphogenesis | 10/18614 | 0.006963761 | 0.032891189 |
| BP | GO:0048865 | stem cell fate commitment | 10/18614 | 0.006963761 | 0.032891189 |
| BP | GO:0060346 | bone trabecula formation | 10/18614 | 0.006963761 | 0.032891189 |
| BP | GO:0060525 | prostate glandular acinus development | 10/18614 | 0.006963761 | 0.032891189 |
| BP | GO:0071377 | cellular response to glucagon stimulus | 10/18614 | 0.006963761 | 0.032891189 |
| BP | GO:0072172 | mesonephric tubule formation | 10/18614 | 0.006963761 | 0.032891189 |
| BP | GO:2000271 | positive regulation of fibroblast apoptotic process | 10/18614 | 0.006963761 | 0.032891189 |
| BP | GO:0030324 | lung development | 186/18614 | 0.007205417 | 0.032891189 |
| BP | GO:0061138 | morphogenesis of a branching epithelium | 188/18614 | 0.007355839 | 0.032891189 |
| BP | GO:0030323 | respiratory tube development | 190/18614 | 0.007507693 | 0.032891189 |
| BP | GO:0003263 | cardioblast proliferation | 11/18614 | 0.007657669 | 0.032891189 |
| BP | GO:0003264 | regulation of cardioblast proliferation | 11/18614 | 0.007657669 | 0.032891189 |
| BP | GO:0007494 | midgut development | 11/18614 | 0.007657669 | 0.032891189 |
| BP | GO:0086100 | endothelin receptor signaling pathway | 11/18614 | 0.007657669 | 0.032891189 |
| BP | GO:0090179 | planar cell polarity pathway involved in neural tube closure | 11/18614 | 0.007657669 | 0.032891189 |
| BP | GO:0048839 | inner ear development | 193/18614 | 0.00773815 | 0.032891189 |
| BP | GO:0009950 | dorsal/ventral axis specification | 12/18614 | 0.00835113 | 0.032891189 |
| BP | GO:0014842 | regulation of skeletal muscle satellite cell proliferation | 12/18614 | 0.00835113 | 0.032891189 |
| BP | GO:0060442 | branching involved in prostate gland morphogenesis | 12/18614 | 0.00835113 | 0.032891189 |
| BP | GO:0060788 | ectodermal placode formation | 12/18614 | 0.00835113 | 0.032891189 |
| BP | GO:0071697 | ectodermal placode morphogenesis | 12/18614 | 0.00835113 | 0.032891189 |
| BP | GO:0090178 | regulation of establishment of planar polarity involved in neural tube closure | 12/18614 | 0.00835113 | 0.032891189 |
| BP | GO:1904338 | regulation of dopaminergic neuron differentiation | 12/18614 | 0.00835113 | 0.032891189 |
| BP | GO:0001763 | morphogenesis of a branching structure | 203/18614 | 0.008529388 | 0.032891189 |
| BP | GO:1902075 | cellular response to salt | 204/18614 | 0.00861045 | 0.032891189 |
| BP | GO:1901605 | alpha-amino acid metabolic process | 206/18614 | 0.008773626 | 0.032891189 |
| BP | GO:1901654 | response to ketone | 208/18614 | 0.008938201 | 0.032891189 |
| BP | GO:0071383 | cellular response to steroid hormone stimulus | 209/18614 | 0.009021012 | 0.032891189 |
| BP | GO:0006563 | L-serine metabolic process | 13/18614 | 0.009044143 | 0.032891189 |
| BP | GO:0034616 | response to laminar fluid shear stress | 13/18614 | 0.009044143 | 0.032891189 |
| BP | GO:0046541 | saliva secretion | 13/18614 | 0.009044143 | 0.032891189 |
| BP | GO:0051451 | myoblast migration | 13/18614 | 0.009044143 | 0.032891189 |
| BP | GO:0061430 | bone trabecula morphogenesis | 13/18614 | 0.009044143 | 0.032891189 |
| BP | GO:0071481 | cellular response to X-ray | 13/18614 | 0.009044143 | 0.032891189 |
| BP | GO:0071696 | ectodermal placode development | 13/18614 | 0.009044143 | 0.032891189 |
| BP | GO:0090177 | establishment of planar polarity involved in neural tube closure | 13/18614 | 0.009044143 | 0.032891189 |
| BP | GO:1900121 | negative regulation of receptor binding | 13/18614 | 0.009044143 | 0.032891189 |
| BP | GO:1901888 | regulation of cell junction assembly | 214/18614 | 0.009440284 | 0.032891189 |
| BP | GO:0006469 | negative regulation of protein kinase activity | 217/18614 | 0.009696005 | 0.032891189 |
| BP | GO:0014841 | skeletal muscle satellite cell proliferation | 14/18614 | 0.009736709 | 0.032891189 |
| BP | GO:0071285 | cellular response to lithium ion | 14/18614 | 0.009736709 | 0.032891189 |
| BP | GO:0072350 | tricarboxylic acid metabolic process | 14/18614 | 0.009736709 | 0.032891189 |
| BP | GO:1902043 | positive regulation of extrinsic apoptotic signaling pathway via death domain receptors | 14/18614 | 0.009736709 | 0.032891189 |
| BP | GO:2000288 | positive regulation of myoblast proliferation | 14/18614 | 0.009736709 | 0.032891189 |
| BP | GO:0043583 | ear development | 221/18614 | 0.010041791 | 0.032891189 |
| BP | GO:0014029 | neural crest formation | 15/18614 | 0.010428829 | 0.032891189 |
| BP | GO:0033689 | negative regulation of osteoblast proliferation | 15/18614 | 0.010428829 | 0.032891189 |
| BP | GO:0043584 | nose development | 15/18614 | 0.010428829 | 0.032891189 |
| BP | GO:0060026 | convergent extension | 15/18614 | 0.010428829 | 0.032891189 |
| BP | GO:0060766 | negative regulation of androgen receptor signaling pathway | 15/18614 | 0.010428829 | 0.032891189 |
| BP | GO:0070863 | positive regulation of protein exit from endoplasmic reticulum | 15/18614 | 0.010428829 | 0.032891189 |
| BP | GO:0071380 | cellular response to prostaglandin E stimulus | 15/18614 | 0.010428829 | 0.032891189 |
| BP | GO:0071599 | otic vesicle development | 15/18614 | 0.010428829 | 0.032891189 |
| BP | GO:0072075 | metanephric mesenchyme development | 15/18614 | 0.010428829 | 0.032891189 |
| BP | GO:2000052 | positive regulation of non-canonical Wnt signaling pathway | 15/18614 | 0.010428829 | 0.032891189 |
| BP | GO:0002573 | myeloid leukocyte differentiation | 227/18614 | 0.010570752 | 0.032891189 |
| BP | GO:0008406 | gonad development | 228/18614 | 0.010660107 | 0.032891189 |
| BP | GO:0045137 | development of primary sexual characteristics | 233/18614 | 0.011111968 | 0.032891189 |
| BP | GO:0006544 | glycine metabolic process | 16/18614 | 0.011120502 | 0.032891189 |
| BP | GO:0014857 | regulation of skeletal muscle cell proliferation | 16/18614 | 0.011120502 | 0.032891189 |
| BP | GO:0021783 | preganglionic parasympathetic fiber development | 16/18614 | 0.011120502 | 0.032891189 |
| BP | GO:0042249 | establishment of planar polarity of embryonic epithelium | 16/18614 | 0.011120502 | 0.032891189 |
| BP | GO:0043508 | negative regulation of JUN kinase activity | 16/18614 | 0.011120502 | 0.032891189 |
| BP | GO:0050862 | positive regulation of T cell receptor signaling pathway | 16/18614 | 0.011120502 | 0.032891189 |
| BP | GO:0061469 | regulation of type B pancreatic cell proliferation | 16/18614 | 0.011120502 | 0.032891189 |
| BP | GO:0090336 | positive regulation of brown fat cell differentiation | 16/18614 | 0.011120502 | 0.032891189 |
| BP | GO:1902033 | regulation of hematopoietic stem cell proliferation | 16/18614 | 0.011120502 | 0.032891189 |
| BP | GO:2000095 | regulation of Wnt signaling pathway, planar cell polarity pathway | 16/18614 | 0.011120502 | 0.032891189 |
| BP | GO:0060560 | developmental growth involved in morphogenesis | 234/18614 | 0.011203355 | 0.032891189 |
| BP | GO:0010466 | negative regulation of peptidase activity | 236/18614 | 0.011387141 | 0.032891189 |
| BP | GO:0033673 | negative regulation of kinase activity | 239/18614 | 0.011665342 | 0.032891189 |
| BP | GO:0009084 | glutamine family amino acid biosynthetic process | 17/18614 | 0.011811728 | 0.032891189 |
| BP | GO:0030322 | stabilization of membrane potential | 17/18614 | 0.011811728 | 0.032891189 |
| BP | GO:0050872 | white fat cell differentiation | 17/18614 | 0.011811728 | 0.032891189 |
| BP | GO:0061548 | ganglion development | 17/18614 | 0.011811728 | 0.032891189 |
| BP | GO:0072189 | ureter development | 17/18614 | 0.011811728 | 0.032891189 |
| BP | GO:1904948 | midbrain dopaminergic neuron differentiation | 17/18614 | 0.011811728 | 0.032891189 |
| BP | GO:0048863 | stem cell differentiation | 247/18614 | 0.012421922 | 0.033168107 |
| BP | GO:0046851 | negative regulation of bone remodeling | 18/18614 | 0.012502509 | 0.033168107 |
| BP | GO:0048486 | parasympathetic nervous system development | 18/18614 | 0.012502509 | 0.033168107 |
| BP | GO:0060602 | branch elongation of an epithelium | 18/18614 | 0.012502509 | 0.033168107 |
| BP | GO:0071391 | cellular response to estrogen stimulus | 18/18614 | 0.012502509 | 0.033168107 |
| BP | GO:0090190 | positive regulation of branching involved in ureteric bud morphogenesis | 18/18614 | 0.012502509 | 0.033168107 |
| BP | GO:2000136 | regulation of cell proliferation involved in heart morphogenesis | 18/18614 | 0.012502509 | 0.033168107 |
| BP | GO:0001649 | osteoblast differentiation | 250/18614 | 0.012711124 | 0.033495226 |
| BP | GO:0061323 | cell proliferation involved in heart morphogenesis | 19/18614 | 0.013192844 | 0.034067497 |
| BP | GO:0072074 | kidney mesenchyme development | 19/18614 | 0.013192844 | 0.034067497 |
| BP | GO:0048762 | mesenchymal cell differentiation | 255/18614 | 0.013199726 | 0.034067497 |
| BP | GO:0006525 | arginine metabolic process | 20/18614 | 0.013882733 | 0.034067497 |
| BP | GO:0042474 | middle ear morphogenesis | 20/18614 | 0.013882733 | 0.034067497 |
| BP | GO:0043586 | tongue development | 20/18614 | 0.013882733 | 0.034067497 |
| BP | GO:0070314 | G1 to G0 transition | 20/18614 | 0.013882733 | 0.034067497 |
| BP | GO:0071379 | cellular response to prostaglandin stimulus | 20/18614 | 0.013882733 | 0.034067497 |
| BP | GO:0072079 | nephron tubule formation | 20/18614 | 0.013882733 | 0.034067497 |
| BP | GO:2000269 | regulation of fibroblast apoptotic process | 20/18614 | 0.013882733 | 0.034067497 |
| BP | GO:2001014 | regulation of skeletal muscle cell differentiation | 20/18614 | 0.013882733 | 0.034067497 |
| BP | GO:0009070 | serine family amino acid biosynthetic process | 21/18614 | 0.014572178 | 0.03467574 |
| BP | GO:0014856 | skeletal muscle cell proliferation | 21/18614 | 0.014572178 | 0.03467574 |
| BP | GO:0033762 | response to glucagon | 21/18614 | 0.014572178 | 0.03467574 |
| BP | GO:0071498 | cellular response to fluid shear stress | 21/18614 | 0.014572178 | 0.03467574 |
| BP | GO:0090189 | regulation of branching involved in ureteric bud morphogenesis | 21/18614 | 0.014572178 | 0.03467574 |
| BP | GO:0050708 | regulation of protein secretion | 275/18614 | 0.015235634 | 0.035880359 |
| BP | GO:0034104 | negative regulation of tissue remodeling | 22/18614 | 0.015261177 | 0.035880359 |
| BP | GO:0051348 | negative regulation of transferase activity | 279/18614 | 0.015658265 | 0.03619866 |
| BP | GO:0045165 | cell fate commitment | 281/18614 | 0.015871489 | 0.03619866 |
| BP | GO:0002070 | epithelial cell maturation | 23/18614 | 0.015949731 | 0.03619866 |
| BP | GO:0019835 | cytolysis | 23/18614 | 0.015949731 | 0.03619866 |
| BP | GO:0071305 | cellular response to vitamin D | 23/18614 | 0.015949731 | 0.03619866 |
| BP | GO:1900120 | regulation of receptor binding | 23/18614 | 0.015949731 | 0.03619866 |
| BP | GO:0007548 | sex differentiation | 283/18614 | 0.016085981 | 0.03620408 |
| BP | GO:0010721 | negative regulation of cell development | 286/18614 | 0.01641009 | 0.03620408 |
| BP | GO:0001759 | organ induction | 24/18614 | 0.016637841 | 0.03620408 |
| BP | GO:0002053 | positive regulation of mesenchymal cell proliferation | 24/18614 | 0.016637841 | 0.03620408 |
| BP | GO:0034695 | response to prostaglandin E | 24/18614 | 0.016637841 | 0.03620408 |
| BP | GO:0090103 | cochlea morphogenesis | 24/18614 | 0.016637841 | 0.03620408 |
| BP | GO:0090335 | regulation of brown fat cell differentiation | 24/18614 | 0.016637841 | 0.03620408 |
| BP | GO:0006520 | amino acid metabolic process | 290/18614 | 0.016846642 | 0.03620408 |
| BP | GO:0048562 | embryonic organ morphogenesis | 294/18614 | 0.017288205 | 0.03620408 |
| BP | GO:0006706 | steroid catabolic process | 25/18614 | 0.017325507 | 0.03620408 |
| BP | GO:0010226 | response to lithium ion | 25/18614 | 0.017325507 | 0.03620408 |
| BP | GO:2000291 | regulation of myoblast proliferation | 25/18614 | 0.017325507 | 0.03620408 |
| BP | GO:0006541 | glutamine metabolic process | 26/18614 | 0.018012729 | 0.03620408 |
| BP | GO:0044346 | fibroblast apoptotic process | 26/18614 | 0.018012729 | 0.03620408 |
| BP | GO:0048745 | smooth muscle tissue development | 26/18614 | 0.018012729 | 0.03620408 |
| BP | GO:0050857 | positive regulation of antigen receptor-mediated signaling pathway | 26/18614 | 0.018012729 | 0.03620408 |
| BP | GO:0051894 | positive regulation of focal adhesion assembly | 26/18614 | 0.018012729 | 0.03620408 |
| BP | GO:0060343 | trabecula formation | 26/18614 | 0.018012729 | 0.03620408 |
| BP | GO:0060740 | prostate gland epithelium morphogenesis | 26/18614 | 0.018012729 | 0.03620408 |
| BP | GO:0070102 | interleukin-6-mediated signaling pathway | 26/18614 | 0.018012729 | 0.03620408 |
| BP | GO:2000050 | regulation of non-canonical Wnt signaling pathway | 26/18614 | 0.018012729 | 0.03620408 |
| BP | GO:2000647 | negative regulation of stem cell proliferation | 26/18614 | 0.018012729 | 0.03620408 |
| BP | GO:0048608 | reproductive structure development | 301/18614 | 0.018072921 | 0.03620408 |
| BP | GO:0061458 | reproductive system development | 305/18614 | 0.01852813 | 0.036894558 |
| BP | GO:0070861 | regulation of protein exit from endoplasmic reticulum | 27/18614 | 0.018699507 | 0.036894558 |
| BP | GO:0071549 | cellular response to dexamethasone stimulus | 27/18614 | 0.018699507 | 0.036894558 |
| BP | GO:0051222 | positive regulation of protein transport | 309/18614 | 0.018988251 | 0.037276934 |
| BP | GO:0060512 | prostate gland morphogenesis | 28/18614 | 0.019385842 | 0.037495043 |
| BP | GO:0060765 | regulation of androgen receptor signaling pathway | 28/18614 | 0.019385842 | 0.037495043 |
| BP | GO:1903779 | regulation of cardiac conduction | 28/18614 | 0.019385842 | 0.037495043 |
| BP | GO:0003156 | regulation of animal organ formation | 29/18614 | 0.020071734 | 0.038071481 |
| BP | GO:0030878 | thyroid gland development | 29/18614 | 0.020071734 | 0.038071481 |
| BP | GO:0044342 | type B pancreatic cell proliferation | 29/18614 | 0.020071734 | 0.038071481 |
| BP | GO:0060037 | pharyngeal system development | 29/18614 | 0.020071734 | 0.038071481 |
| BP | GO:0060485 | mesenchyme development | 320/18614 | 0.020278693 | 0.038279112 |
| BP | GO:0008202 | steroid metabolic process | 323/18614 | 0.020636972 | 0.038611831 |
| BP | GO:0021602 | cranial nerve morphogenesis | 30/18614 | 0.020757183 | 0.038611831 |
| BP | GO:0043032 | positive regulation of macrophage activation | 30/18614 | 0.020757183 | 0.038611831 |
| BP | GO:1904951 | positive regulation of establishment of protein localization | 325/18614 | 0.020877324 | 0.038611831 |
| BP | GO:0046394 | carboxylic acid biosynthetic process | 328/18614 | 0.021240091 | 0.038611831 |
| BP | GO:0006884 | cell volume homeostasis | 31/18614 | 0.021442189 | 0.038611831 |
| BP | GO:0010464 | regulation of mesenchymal cell proliferation | 31/18614 | 0.021442189 | 0.038611831 |
| BP | GO:0033688 | regulation of osteoblast proliferation | 31/18614 | 0.021442189 | 0.038611831 |
| BP | GO:0150117 | positive regulation of cell-substrate junction organization | 31/18614 | 0.021442189 | 0.038611831 |
| BP | GO:0045861 | negative regulation of proteolysis | 330/18614 | 0.021483424 | 0.038611831 |
| BP | GO:0016053 | organic acid biosynthetic process | 331/18614 | 0.021605536 | 0.038611831 |
| BP | GO:0060562 | epithelial tube morphogenesis | 334/18614 | 0.021973648 | 0.038611831 |
| BP | GO:0010165 | response to X-ray | 32/18614 | 0.022126753 | 0.038611831 |
| BP | GO:0034694 | response to prostaglandin | 32/18614 | 0.022126753 | 0.038611831 |
| BP | GO:0045671 | negative regulation of osteoclast differentiation | 32/18614 | 0.022126753 | 0.038611831 |
| BP | GO:0045736 | negative regulation of cyclin-dependent protein serine/threonine kinase activity | 32/18614 | 0.022126753 | 0.038611831 |
| BP | GO:0071295 | cellular response to vitamin | 32/18614 | 0.022126753 | 0.038611831 |
| BP | GO:0048545 | response to steroid hormone | 339/18614 | 0.022593065 | 0.038759261 |
| BP | GO:0048147 | negative regulation of fibroblast proliferation | 33/18614 | 0.022810875 | 0.038759261 |
| BP | GO:0048665 | neuron fate specification | 33/18614 | 0.022810875 | 0.038759261 |
| BP | GO:0090183 | regulation of kidney development | 33/18614 | 0.022810875 | 0.038759261 |
| BP | GO:1904030 | negative regulation of cyclin-dependent protein kinase activity | 33/18614 | 0.022810875 | 0.038759261 |
| BP | GO:0001933 | negative regulation of protein phosphorylation | 341/18614 | 0.022842887 | 0.038759261 |
| BP | GO:0051346 | negative regulation of hydrolase activity | 345/18614 | 0.023346034 | 0.038759261 |
| BP | GO:0034405 | response to fluid shear stress | 34/18614 | 0.023494555 | 0.038759261 |
| BP | GO:0045577 | regulation of B cell differentiation | 34/18614 | 0.023494555 | 0.038759261 |
| BP | GO:0050869 | negative regulation of B cell activation | 34/18614 | 0.023494555 | 0.038759261 |
| BP | GO:0051450 | myoblast proliferation | 34/18614 | 0.023494555 | 0.038759261 |
| BP | GO:0060914 | heart formation | 34/18614 | 0.023494555 | 0.038759261 |
| BP | GO:0110110 | positive regulation of animal organ morphogenesis | 34/18614 | 0.023494555 | 0.038759261 |
| BP | GO:0007517 | muscle organ development | 351/18614 | 0.024109469 | 0.038905595 |
| BP | GO:0018149 | peptide cross-linking | 35/18614 | 0.024177793 | 0.038905595 |
| BP | GO:0031128 | developmental induction | 35/18614 | 0.024177793 | 0.038905595 |
| BP | GO:0060416 | response to growth hormone | 35/18614 | 0.024177793 | 0.038905595 |
| BP | GO:0071398 | cellular response to fatty acid | 35/18614 | 0.024177793 | 0.038905595 |
| BP | GO:0071425 | hematopoietic stem cell proliferation | 35/18614 | 0.024177793 | 0.038905595 |
| BP | GO:0033687 | osteoblast proliferation | 36/18614 | 0.02486059 | 0.039518433 |
| BP | GO:0060218 | hematopoietic stem cell differentiation | 36/18614 | 0.02486059 | 0.039518433 |
| BP | GO:1905332 | positive regulation of morphogenesis of an epithelium | 36/18614 | 0.02486059 | 0.039518433 |
| BP | GO:0010092 | specification of animal organ identity | 37/18614 | 0.025542947 | 0.03979749 |
| BP | GO:0033144 | negative regulation of intracellular steroid hormone receptor signaling pathway | 37/18614 | 0.025542947 | 0.03979749 |
| BP | GO:0033280 | response to vitamin D | 37/18614 | 0.025542947 | 0.03979749 |
| BP | GO:0035909 | aorta morphogenesis | 37/18614 | 0.025542947 | 0.03979749 |
| BP | GO:0048741 | skeletal muscle fiber development | 37/18614 | 0.025542947 | 0.03979749 |
| BP | GO:0045862 | positive regulation of proteolysis | 365/18614 | 0.025931033 | 0.040221519 |
| BP | GO:0010038 | response to metal ion | 367/18614 | 0.026195806 | 0.040221519 |
| BP | GO:0045880 | positive regulation of smoothened signaling pathway | 38/18614 | 0.026224862 | 0.040221519 |
| BP | GO:0046676 | negative regulation of insulin secretion | 38/18614 | 0.026224862 | 0.040221519 |
| BP | GO:0071900 | regulation of protein serine/threonine kinase activity | 369/18614 | 0.026461707 | 0.040426855 |
| BP | GO:0030279 | negative regulation of ossification | 39/18614 | 0.026906337 | 0.040612367 |
| BP | GO:0030851 | granulocyte differentiation | 39/18614 | 0.026906337 | 0.040612367 |
| BP | GO:0046329 | negative regulation of JNK cascade | 39/18614 | 0.026906337 | 0.040612367 |
| BP | GO:0009306 | protein secretion | 373/18614 | 0.02699688 | 0.040612367 |
| BP | GO:0035592 | establishment of protein localization to extracellular region | 374/18614 | 0.027131373 | 0.040658908 |
| BP | GO:0009069 | serine family amino acid metabolic process | 40/18614 | 0.027587371 | 0.041029065 |
| BP | GO:0071392 | cellular response to estradiol stimulus | 40/18614 | 0.027587371 | 0.041029065 |
| BP | GO:0071692 | protein localization to extracellular region | 382/18614 | 0.02821734 | 0.041080467 |
| BP | GO:0014904 | myotube cell development | 41/18614 | 0.028267966 | 0.041080467 |
| BP | GO:0032941 | secretion by tissue | 41/18614 | 0.028267966 | 0.041080467 |
| BP | GO:0140467 | integrated stress response signaling | 41/18614 | 0.028267966 | 0.041080467 |
| BP | GO:1901381 | positive regulation of potassium ion transmembrane transport | 41/18614 | 0.028267966 | 0.041080467 |
| BP | GO:0042326 | negative regulation of phosphorylation | 383/18614 | 0.028354333 | 0.041080467 |
| BP | GO:1902074 | response to salt | 383/18614 | 0.028354333 | 0.041080467 |
| BP | GO:0045429 | positive regulation of nitric oxide biosynthetic process | 42/18614 | 0.028948121 | 0.041481556 |
| BP | GO:0071354 | cellular response to interleukin-6 | 42/18614 | 0.028948121 | 0.041481556 |
| BP | GO:0071542 | dopaminergic neuron differentiation | 42/18614 | 0.028948121 | 0.041481556 |
| BP | GO:0010463 | mesenchymal cell proliferation | 43/18614 | 0.029627836 | 0.04169471 |
| BP | GO:0010719 | negative regulation of epithelial to mesenchymal transition | 43/18614 | 0.029627836 | 0.04169471 |
| BP | GO:0031670 | cellular response to nutrient | 43/18614 | 0.029627836 | 0.04169471 |
| BP | GO:0071548 | response to dexamethasone | 43/18614 | 0.029627836 | 0.04169471 |
| BP | GO:0090278 | negative regulation of peptide hormone secretion | 43/18614 | 0.029627836 | 0.04169471 |
| BP | GO:0002792 | negative regulation of peptide secretion | 44/18614 | 0.030307112 | 0.042196913 |
| BP | GO:0050856 | regulation of T cell receptor signaling pathway | 44/18614 | 0.030307112 | 0.042196913 |
| BP | GO:1904407 | positive regulation of nitric oxide metabolic process | 44/18614 | 0.030307112 | 0.042196913 |
| BP | GO:0048483 | autonomic nervous system development | 45/18614 | 0.03098595 | 0.042838248 |
| BP | GO:0070741 | response to interleukin-6 | 45/18614 | 0.03098595 | 0.042838248 |
| BP | GO:0030521 | androgen receptor signaling pathway | 47/18614 | 0.032342308 | 0.044247878 |
| BP | GO:0061383 | trabecula morphogenesis | 47/18614 | 0.032342308 | 0.044247878 |
| BP | GO:1903706 | regulation of hemopoiesis | 415/18614 | 0.03288159 | 0.044247878 |
| BP | GO:0006953 | acute-phase response | 48/18614 | 0.03301983 | 0.044247878 |
| BP | GO:0014075 | response to amine | 48/18614 | 0.03301983 | 0.044247878 |
| BP | GO:0030850 | prostate gland development | 48/18614 | 0.03301983 | 0.044247878 |
| BP | GO:0032527 | protein exit from endoplasmic reticulum | 48/18614 | 0.03301983 | 0.044247878 |
| BP | GO:0043268 | positive regulation of potassium ion transport | 48/18614 | 0.03301983 | 0.044247878 |
| BP | GO:0048538 | thymus development | 48/18614 | 0.03301983 | 0.044247878 |
| BP | GO:0050678 | regulation of epithelial cell proliferation | 419/18614 | 0.033466725 | 0.04448182 |
| BP | GO:0007528 | neuromuscular junction development | 49/18614 | 0.033696914 | 0.04448182 |
| BP | GO:0010043 | response to zinc ion | 49/18614 | 0.033696914 | 0.04448182 |
| BP | GO:0046850 | regulation of bone remodeling | 49/18614 | 0.033696914 | 0.04448182 |
| BP | GO:0030099 | myeloid cell differentiation | 421/18614 | 0.033760867 | 0.04448182 |
| BP | GO:0003002 | regionalization | 422/18614 | 0.033908329 | 0.044526692 |
| BP | GO:0048546 | digestive tract morphogenesis | 50/18614 | 0.034373561 | 0.044689223 |
| BP | GO:0090102 | cochlea development | 50/18614 | 0.034373561 | 0.044689223 |
| BP | GO:1902041 | regulation of extrinsic apoptotic signaling pathway via death domain receptors | 50/18614 | 0.034373561 | 0.044689223 |
| BP | GO:0001503 | ossification | 429/18614 | 0.034947853 | 0.044972701 |
| BP | GO:0032873 | negative regulation of stress-activated MAPK cascade | 51/18614 | 0.03504977 | 0.044972701 |
| BP | GO:0070303 | negative regulation of stress-activated protein kinase signaling cascade | 51/18614 | 0.03504977 | 0.044972701 |
| BP | GO:2001238 | positive regulation of extrinsic apoptotic signaling pathway | 51/18614 | 0.03504977 | 0.044972701 |
| BP | GO:0002762 | negative regulation of myeloid leukocyte differentiation | 52/18614 | 0.035725542 | 0.045542129 |
| BP | GO:0051496 | positive regulation of stress fiber assembly | 52/18614 | 0.035725542 | 0.045542129 |
| BP | GO:0060071 | Wnt signaling pathway, planar cell polarity pathway | 53/18614 | 0.036400877 | 0.046067401 |
| BP | GO:0045936 | negative regulation of phosphate metabolic process | 439/18614 | 0.036454809 | 0.046067401 |
| BP | GO:0009410 | response to xenobiotic stimulus | 440/18614 | 0.03660691 | 0.046067401 |
| BP | GO:0010563 | negative regulation of phosphorus metabolic process | 440/18614 | 0.03660691 | 0.046067401 |
| BP | GO:0071320 | cellular response to cAMP | 54/18614 | 0.037075775 | 0.046360255 |
| BP | GO:0071385 | cellular response to glucocorticoid stimulus | 54/18614 | 0.037075775 | 0.046360255 |
| BP | GO:0034329 | cell junction assembly | 444/18614 | 0.037217856 | 0.046390176 |
| BP | GO:0006869 | lipid transport | 447/18614 | 0.03767872 | 0.046815998 |
| BP | GO:0001954 | positive regulation of cell-matrix adhesion | 56/18614 | 0.038424263 | 0.047591732 |
| BP | GO:0006816 | calcium ion transport | 455/18614 | 0.038918736 | 0.048052594 |
| BP | GO:0090175 | regulation of establishment of planar polarity | 57/18614 | 0.039097853 | 0.048122419 |
| BP | GO:0030225 | macrophage differentiation | 58/18614 | 0.039771007 | 0.048645961 |
| BP | GO:0050732 | negative regulation of peptidyl-tyrosine phosphorylation | 58/18614 | 0.039771007 | 0.048645961 |
| BP | GO:0007389 | pattern specification process | 467/18614 | 0.040808547 | 0.049368331 |
| BP | GO:1903829 | positive regulation of protein localization | 468/18614 | 0.040967628 | 0.049368331 |
| BP | GO:0001658 | branching involved in ureteric bud morphogenesis | 60/18614 | 0.041116011 | 0.049368331 |
| BP | GO:0021545 | cranial nerve development | 60/18614 | 0.041116011 | 0.049368331 |
| BP | GO:0043407 | negative regulation of MAP kinase activity | 60/18614 | 0.041116011 | 0.049368331 |
| BP | GO:0045620 | negative regulation of lymphocyte differentiation | 60/18614 | 0.041116011 | 0.049368331 |
| BP | GO:0045668 | negative regulation of osteoblast differentiation | 61/18614 | 0.04178786 | 0.050022053 |
| BP | GO:0031667 | response to nutrient levels | 477/18614 | 0.042410296 | 0.050062619 |
| BP | GO:0032233 | positive regulation of actin filament bundle assembly | 62/18614 | 0.042459275 | 0.050062619 |
| BP | GO:0043030 | regulation of macrophage activation | 62/18614 | 0.042459275 | 0.050062619 |
| BP | GO:0043506 | regulation of JUN kinase activity | 62/18614 | 0.042459275 | 0.050062619 |
| BP | GO:0045428 | regulation of nitric oxide biosynthetic process | 62/18614 | 0.042459275 | 0.050062619 |
| BP | GO:0035904 | aorta development | 63/18614 | 0.043130255 | 0.050399703 |
| BP | GO:0070059 | intrinsic apoptotic signaling pathway in response to endoplasmic reticulum stress | 63/18614 | 0.043130255 | 0.050399703 |
| BP | GO:0070542 | response to fatty acid | 63/18614 | 0.043130255 | 0.050399703 |
| BP | GO:0019731 | antibacterial humoral response | 64/18614 | 0.043800802 | 0.050581111 |
| BP | GO:0051893 | regulation of focal adhesion assembly | 64/18614 | 0.043800802 | 0.050581111 |
| BP | GO:0071384 | cellular response to corticosteroid stimulus | 64/18614 | 0.043800802 | 0.050581111 |
| BP | GO:0090109 | regulation of cell-substrate junction assembly | 64/18614 | 0.043800802 | 0.050581111 |
| BP | GO:0002683 | negative regulation of immune system process | 487/18614 | 0.044036124 | 0.050610682 |
| BP | GO:0050673 | epithelial cell proliferation | 488/18614 | 0.044200017 | 0.050610682 |
| BP | GO:0046888 | negative regulation of hormone secretion | 65/18614 | 0.044470914 | 0.050610682 |
| BP | GO:0050854 | regulation of antigen receptor-mediated signaling pathway | 65/18614 | 0.044470914 | 0.050610682 |
| BP | GO:0080164 | regulation of nitric oxide metabolic process | 65/18614 | 0.044470914 | 0.050610682 |
| BP | GO:0001655 | urogenital system development | 66/18614 | 0.045140593 | 0.05092995 |
| BP | GO:0001756 | somitogenesis | 66/18614 | 0.045140593 | 0.05092995 |
| BP | GO:0060675 | ureteric bud morphogenesis | 66/18614 | 0.045140593 | 0.05092995 |
| BP | GO:0010876 | lipid localization | 497/18614 | 0.045685674 | 0.051389684 |
| BP | GO:0072171 | mesonephric tubule morphogenesis | 67/18614 | 0.045809839 | 0.051389684 |
| BP | GO:0035019 | somatic stem cell population maintenance | 68/18614 | 0.046478652 | 0.051405596 |
| BP | GO:0035914 | skeletal muscle cell differentiation | 68/18614 | 0.046478652 | 0.051405596 |
| BP | GO:0045670 | regulation of osteoclast differentiation | 68/18614 | 0.046478652 | 0.051405596 |
| BP | GO:0048645 | animal organ formation | 68/18614 | 0.046478652 | 0.051405596 |
| BP | GO:0048663 | neuron fate commitment | 68/18614 | 0.046478652 | 0.051405596 |
| BP | GO:0150116 | regulation of cell-substrate junction organization | 69/18614 | 0.047147032 | 0.051998353 |
| BP | GO:0030514 | negative regulation of BMP signaling pathway | 70/18614 | 0.04781498 | 0.052440422 |
| BP | GO:0043627 | response to estrogen | 70/18614 | 0.04781498 | 0.052440422 |
| BP | GO:0033143 | regulation of intracellular steroid hormone receptor signaling pathway | 72/18614 | 0.049149579 | 0.053753974 |
| BP | GO:0035567 | non-canonical Wnt signaling pathway | 73/18614 | 0.04981623 | 0.054331737 |
| CC | GO:0031094 | platelet dense tubular network | 11/19518 | 0.007304085 | 0.061916956 |
| CC | GO:1904090 | peptidase inhibitor complex | 11/19518 | 0.007304085 | 0.061916956 |
| CC | GO:1990531 | phospholipid-translocating ATPase complex | 14/19518 | 0.009287543 | 0.061916956 |
| CC | GO:0033017 | sarcoplasmic reticulum membrane | 42/19518 | 0.027624217 | 0.106057003 |
| CC | GO:1990351 | transporter complex | 418/19518 | 0.030534945 | 0.106057003 |
| CC | GO:0001533 | cornified envelope | 59/19518 | 0.038603819 | 0.106057003 |
| CC | GO:0043025 | neuronal cell body | 500/19518 | 0.042387375 | 0.106057003 |
| CC | GO:0016529 | sarcoplasmic reticulum | 73/19518 | 0.047559792 | 0.106057003 |
| MF | GO:0061134 | peptidase regulator activity | 230/18369 | 0.000636874 | 0.016283912 |
| MF | GO:0004869 | cysteine-type endopeptidase inhibitor activity | 54/18369 | 0.00075462 | 0.016283912 |
| MF | GO:0005496 | steroid binding | 99/18369 | 0.002508504 | 0.035483066 |
| MF | GO:0016616 | oxidoreductase activity, acting on the CH-OH group of donors, NAD or NADP as acceptor | 123/18369 | 0.003839588 | 0.035483066 |
| MF | GO:0016614 | oxidoreductase activity, acting on CH-OH group of donors | 133/18369 | 0.004472565 | 0.035483066 |
| MF | GO:0004866 | endopeptidase inhibitor activity | 173/18369 | 0.007449961 | 0.035483066 |
| MF | GO:0015643 | toxic substance binding | 10/18369 | 0.007597305 | 0.035483066 |
| MF | GO:0030414 | peptidase inhibitor activity | 180/18369 | 0.008042368 | 0.035483066 |
| MF | GO:0061135 | endopeptidase regulator activity | 187/18369 | 0.008655498 | 0.035483066 |
| MF | GO:0015269 | calcium-activated potassium channel activity | 12/18369 | 0.009110319 | 0.035483066 |
| MF | GO:0097677 | STAT family protein binding | 12/18369 | 0.009110319 | 0.035483066 |
| MF | GO:0016208 | AMP binding | 13/18369 | 0.009866023 | 0.035483066 |
| MF | GO:0140327 | flippase activity | 16/18369 | 0.012129925 | 0.040269387 |
| MF | GO:0015662 | P-type ion transporter activity | 20/18369 | 0.015140984 | 0.043502005 |
| MF | GO:0008656 | cysteine-type endopeptidase activator activity involved in apoptotic process | 21/18369 | 0.015892416 | 0.043502005 |
| MF | GO:0005227 | calcium activated cation channel activity | 24/18369 | 0.018143519 | 0.043502005 |
| MF | GO:0016505 | peptidase activator activity involved in apoptotic process | 24/18369 | 0.018143519 | 0.043502005 |
| MF | GO:0140358 | P-type transmembrane transporter activity | 24/18369 | 0.018143519 | 0.043502005 |
| MF | GO:0140326 | ATPase-coupled intramembrane lipid transporter activity | 27/18369 | 0.02038984 | 0.046314872 |
| MF | GO:0033764 | steroid dehydrogenase activity, acting on the CH-OH group of donors, NAD or NADP as acceptor | 31/18369 | 0.023377511 | 0.049576308 |
| MF | GO:0017147 | Wnt-protein binding | 32/18369 | 0.024123106 | 0.049576308 |
| MF | GO:0016229 | steroid dehydrogenase activity | 35/18369 | 0.02635672 | 0.051613384 |
| MF | GO:0005109 | frizzled binding | 37/18369 | 0.027843158 | 0.051613384 |
| MF | GO:0004857 | enzyme inhibitor activity | 359/18369 | 0.029682933 | 0.051613384 |
| MF | GO:0140303 | intramembrane lipid transporter activity | 42/18369 | 0.031550037 | 0.051613384 |
| MF | GO:0001223 | transcription coactivator binding | 43/18369 | 0.032289837 | 0.051613384 |
| MF | GO:0043028 | cysteine-type endopeptidase regulator activity involved in apoptotic process | 43/18369 | 0.032289837 | 0.051613384 |
| MF | GO:0016879 | ligase activity, forming carbon-nitrogen bonds | 48/18369 | 0.035980967 | 0.053487496 |
| MF | GO:0016504 | peptidase activator activity | 50/18369 | 0.037453756 | 0.053487496 |
| MF | GO:0051287 | NAD binding | 52/18369 | 0.038924455 | 0.053487496 |
| MF | GO:0019829 | ATPase-coupled monoatomic cation transmembrane transporter activity | 53/18369 | 0.039659021 | 0.053487496 |
| MF | GO:0050661 | NADP binding | 53/18369 | 0.039659021 | 0.053487496 |
| MF | GO:0016597 | amino acid binding | 55/18369 | 0.041126591 | 0.053555138 |
| MF | GO:0046873 | metal ion transmembrane transporter activity | 435/18369 | 0.042190999 | 0.053555138 |
| MF | GO:0005548 | phospholipid transporter activity | 64/18369 | 0.047704919 | 0.055530498 |
| MF | GO:0001228 | DNA-binding transcription activator activity, RNA polymerase II-specific | 468/18369 | 0.048151046 | 0.055530498 |
| MF | GO:0030145 | manganese ion binding | 65/18369 | 0.048433252 | 0.055530498 |
| MF | GO:0001216 | DNA-binding transcription activator activity | 472/18369 | 0.048893927 | 0.055530498 |

**Table S4b. Analysis of KEGG.**

| ID | Description | BgRatio | pvalue | qvalue |
| --- | --- | --- | --- | --- |
| hsa01230 | Biosynthesis of amino acids | 75/8843 | 0.001922823 | 0.056672683 |
| hsa05202 | Transcriptional misregulation in cancer | 193/8843 | 0.012168313 | 0.142717457 |
| hsa00220 | Arginine biosynthesis | 23/8843 | 0.020627077 | 0.142717457 |
| hsa00250 | Alanine, aspartate and glutamate metabolism | 37/8843 | 0.032999569 | 0.142717457 |
| hsa00260 | Glycine, serine and threonine metabolism | 41/8843 | 0.03650935 | 0.142717457 |
| hsa00270 | Cysteine and methionine metabolism | 52/8843 | 0.046103838 | 0.142717457 |

# Appendix 5

**Gene regulatory networks**

**Table S5.Gene regulatory networks**

| Entity 1 | Entity 2 | Weight | Network group |
| --- | --- | --- | --- |
| CEBPA | IRX5 | 0.013820167 | Co-expression |
| CSTA | HSD11B1 | 0.014110892 | Co-expression |
| PHGDH | WFDC2 | 0.010941287 | Co-expression |
| PHGDH | ASS1 | 0.016998895 | Co-expression |
| IL12RB2 | WFDC2 | 0.036035333 | Co-expression |
| IL12RB2 | ASS1 | 0.017292699 | Co-expression |
| IL12RB2 | PHGDH | 0.012384675 | Co-expression |
| BCHE | HSD11B1 | 0.014333956 | Co-expression |
| BCHE | CSTA | 0.0251102 | Co-expression |
| SLC1A5 | ASS1 | 0.018621964 | Co-expression |
| SLC1A5 | PHGDH | 0.019503722 | Co-expression |
| RBP1 | WFDC2 | 0.01336355 | Co-expression |
| RBP1 | ASS1 | 0.014159819 | Co-expression |
| RBP1 | PHGDH | 0.015939545 | Co-expression |
| RBP1 | IL12RB2 | 0.011031509 | Co-expression |
| BRINP3 | MPPED2 | 0.039103784 | Co-expression |
| BRINP3 | KCNN4 | 0.011244562 | Co-expression |
| NR1H3 | MMD | 0.016909484 | Co-expression |
| KCND2 | MPPED2 | 0.018708752 | Co-expression |
| KCND2 | WFDC2 | 0.02543155 | Co-expression |
| KCND2 | IL12RB2 | 0.018193323 | Co-expression |
| CD70 | HSD11B1 | 0.017292283 | Co-expression |
| CD70 | CSTA | 0.027706964 | Co-expression |
| CD70 | BCHE | 0.024498595 | Co-expression |
| BAMBI | WFDC2 | 0.028122365 | Co-expression |
| BAMBI | IL12RB2 | 0.023765886 | Co-expression |
| FAT1 | WFDC2 | 0.0221116 | Co-expression |
| FAT1 | SFRP1 | 0.026240494 | Co-expression |
| FAT1 | IL12RB2 | 0.017112358 | Co-expression |
| FAT1 | BAMBI | 0.019187013 | Co-expression |
| DLG3 | CSTA | 0.015387116 | Co-expression |
| ASNS | ASS1 | 0.012305662 | Co-expression |
| ASNS | PHGDH | 0.016027262 | Co-expression |
| ASNS | SLC1A5 | 0.027811335 | Co-expression |
| TMC6 | WFDC2 | 0.017216496 | Co-expression |
| TMC6 | ASS1 | 0.013012344 | Co-expression |
| TMC6 | PHGDH | 0.012752964 | Co-expression |
| TMC6 | IL12RB2 | 0.020601705 | Co-expression |
| TMC6 | RBP1 | 0.014074856 | Co-expression |
| PEG3 | HSD11B1 | 0.013072332 | Co-expression |
| PEG3 | SFRP1 | 0.021718165 | Co-expression |
| PEG3 | CSTA | 0.022631243 | Co-expression |
| PEG3 | BCHE | 0.020507976 | Co-expression |
| PEG3 | FAT1 | 0.016752582 | Co-expression |
| PHLDA2 | WFDC2 | 0.018881332 | Co-expression |
| PHLDA2 | ASS1 | 0.01454355 | Co-expression |
| PHLDA2 | IL12RB2 | 0.023349738 | Co-expression |
| PHLDA2 | BAMBI | 0.026761975 | Co-expression |
| SFRP1 | KCNN4 | 0.005719779 | Co-expression |
| CSTA | SIX1 | 0.02497834 | Co-expression |
| PHGDH | KCNN4 | 0.014056684 | Co-expression |
| IL12RB2 | MPPED2 | 0.013666921 | Co-expression |
| BCHE | CSTA | 0.036892958 | Co-expression |
| SLC1A5 | CEBPA | 0.018952595 | Co-expression |
| SLC1A5 | ASS1 | 0.015512892 | Co-expression |
| RBP1 | KCNN4 | 0.018486347 | Co-expression |
| RBP1 | PHGDH | 0.015539736 | Co-expression |
| NR1H3 | HSD11B1 | 0.025421036 | Co-expression |
| CD70 | HSD11B1 | 0.021458965 | Co-expression |
| BAMBI | SIX1 | 0.019770257 | Co-expression |
| TMEM106C | SIX1 | 0.023387743 | Co-expression |
| TMEM106C | CSTA | 0.029496992 | Co-expression |
| DLG3 | SIX1 | 0.020617347 | Co-expression |
| DLG3 | WFDC2 | 0.021470616 | Co-expression |
| ASNS | ASS1 | 0.01660444 | Co-expression |
| ADH5 | SFRP1 | 0.008473956 | Co-expression |
| PEG3 | FAT1 | 0.017845111 | Co-expression |
| PHLDA2 | ASS1 | 0.015499147 | Co-expression |
| MMD | HSD11B1 | 0.016139016 | Co-expression |
| CSTA | HSD11B1 | 0.007617375 | Co-expression |
| CSTA | MMD | 0.006513864 | Co-expression |
| BCHE | PHGDH | 0.007611622 | Co-expression |
| RBP1 | CSTA | 0.003384451 | Co-expression |
| KCND2 | MMD | 0.017945653 | Co-expression |
| KCND2 | CSTA | 0.006198912 | Co-expression |
| KCND2 | RBP1 | 0.006254361 | Co-expression |
| BAMBI | PHGDH | 0.010402375 | Co-expression |
| TMEM106C | FAM46C | 0.019804595 | Co-expression |
| FAT1 | PHGDH | 0.007831599 | Co-expression |
| ASNS | PHGDH | 0.004119569 | Co-expression |
| TMC6 | CSTA | 0.008944733 | Co-expression |
| TMC6 | RBP1 | 0.008006102 | Co-expression |
| PEG3 | ASS1 | 0.002974581 | Co-expression |
| SFRP1 | IRX5 | 0.01196375 | Co-expression |
| ASS1 | CSTA | 0.015733778 | Co-expression |
| ASNS | SLC1A5 | 0.010935986 | Co-expression |
| ADH5 | CCDC25 | 0.01965006 | Co-expression |
| PHLDA2 | SLC1A5 | 0.008119274 | Co-expression |
| ASS1 | SFRP1 | 0.018584535 | Co-expression |
| NR1H3 | SFRP1 | 0.007812331 | Co-expression |
| NR1H3 | ASS1 | 0.00739263 | Co-expression |
| MPPED2 | SIX1 | 0.024566445 | Co-expression |
| SLC1A5 | PHGDH | 0.008901029 | Co-expression |
| NR1H3 | CEBPA | 0.017400289 | Co-expression |
| BAMBI | BCHE | 0.012329642 | Co-expression |
| ASNS | PHGDH | 0.009657901 | Co-expression |
| ASNS | SLC1A5 | 0.007757036 | Co-expression |
| TMC6 | ATP2A3 | 0.01960558 | Co-expression |
| PEG3 | BCHE | 0.012733683 | Co-expression |
| SFRP1 | MMD | 0.01696739 | Co-expression |
| PHGDH | WFDC2 | 0.006157737 | Co-expression |
| SLC1A5 | ATP2A3 | 0.012328786 | Co-expression |
| BRINP3 | KCNN4 | 0.023153614 | Co-expression |
| DLG3 | CSTA | 0.012344128 | Co-expression |
| ADH5 | MPPED2 | 0.026176058 | Co-expression |
| TMC6 | CD70 | 0.007459657 | Co-expression |
| IRX5 | SIX1 | 0.024873164 | Co-expression |
| NR1H3 | CEBPA | 0.022834266 | Co-expression |
| FAT1 | SFRP1 | 0.009633014 | Co-expression |
| PHLDA2 | KCNN4 | 0.027900025 | Co-expression |
| KCNN4 | ATP2A3 | 0.012977362 | Co-expression |
| WFDC2 | KCNN4 | 0.007754214 | Co-expression |
| NR1H3 | CEBPA | 0.014491657 | Co-expression |
| BAMBI | RBP1 | 0.017929954 | Co-expression |
| ADH5 | FAT1 | 0.018315056 | Co-expression |
| PHGDH | IRX5 | 0.014341823 | Co-expression |
| NR1H3 | PHGDH | 0.010974834 | Co-expression |
| BAMBI | MMD | 0.012489243 | Co-expression |
| FAT1 | SFRP1 | 0.01258786 | Co-expression |
| FAT1 | CSTA | 0.010285089 | Co-expression |
| KCNN4 | ATP2A3 | 0.009038953 | Co-expression |
| PHGDH | FAM46C | 0.007463337 | Co-expression |
| SLC1A5 | ATP2A3 | 0.012575746 | Co-expression |
| SLC1A5 | WFDC2 | 0.014387536 | Co-expression |
| BAMBI | WFDC2 | 0.014562954 | Co-expression |
| FAT1 | TMEM106C | 0.019584702 | Co-expression |
| ASNS | FAM46C | 0.006825514 | Co-expression |
| TMC6 | KCNN4 | 0.006458893 | Co-expression |
| PHGDH | IRX5 | 0.01201001 | Co-expression |
| BAMBI | MMD | 0.016898163 | Co-expression |
| FAT1 | MMD | 0.015433438 | Co-expression |
| FAT1 | SLC1A5 | 0.014476 | Co-expression |
| KCNN4 | ATP2A3 | 0.009789888 | Co-expression |
| CD70 | SIX1 | 0.006271673 | Co-expression |
| CD70 | KCNN4 | 0.005141268 | Co-expression |
| PHLDA2 | BAMBI | 0.008298462 | Co-expression |
| MMD | MPPED2 | 0.000691055 | Genetic Interactions |
| CEBPA | SIX1 | 0.000757692 | Genetic Interactions |
| CEBPA | ATP2A3 | 0.000775558 | Genetic Interactions |
| CEBPA | MMD | 0.000449577 | Genetic Interactions |
| SFRP1 | MMD | 0.00057594 | Genetic Interactions |
| ASS1 | MMD | 0.000604168 | Genetic Interactions |
| ASS1 | CEBPA | 0.000626582 | Genetic Interactions |
| PHGDH | CSTA | 0.000883788 | Genetic Interactions |
| IL12RB2 | SFRP1 | 0.001147438 | Genetic Interactions |
| BCHE | SIX1 | 0.000799267 | Genetic Interactions |
| BCHE | MPPED2 | 0.000756018 | Genetic Interactions |
| BCHE | MMD | 0.000474245 | Genetic Interactions |
| BCHE | CEBPA | 0.000491839 | Genetic Interactions |
| BCHE | KCNN4 | 0.00153807 | Genetic Interactions |
| BCHE | PHGDH | 0.000570742 | Genetic Interactions |
| BRINP3 | SIX1 | 0.001125192 | Genetic Interactions |
| BRINP3 | MPPED2 | 0.001064307 | Genetic Interactions |
| BRINP3 | PHGDH | 0.000803479 | Genetic Interactions |
| BRINP3 | BCHE | 0.000730394 | Genetic Interactions |
| KCND2 | FAM46C | 0.000820404 | Genetic Interactions |
| KCND2 | MMD | 0.000488664 | Genetic Interactions |
| KCND2 | CSTA | 0.000827825 | Genetic Interactions |
| KCND2 | ASS1 | 0.000681059 | Genetic Interactions |
| CD70 | CEBPA | 0.003079888 | Genetic Interactions |
| BAMBI | KCND2 | 0.00105387 | Genetic Interactions |
| CCDC25 | ATP2A3 | 0.001345851 | Genetic Interactions |
| ADH5 | PHGDH | 0.00097398 | Genetic Interactions |
| ADH5 | NR1H3 | 0.005002771 | Genetic Interactions |
| PHLDA2 | CEBPA | 0.001377371 | Genetic Interactions |
| PHLDA2 | PHGDH | 0.001598334 | Genetic Interactions |
| PHLDA2 | IL12RB2 | 0.002645956 | Genetic Interactions |
| PHGDH | ASS1 | 0.022711957 | Physical Interactions |
| CENPK | ASS1 | 0.34758112 | Physical Interactions |
| CCDC25 | PHGDH | 0.14619225 | Physical Interactions |
| ASNS | ASS1 | 0.01778589 | Physical Interactions |
| ASNS | PHGDH | 0.019781161 | Physical Interactions |
| ADH5 | PHGDH | 0.11525235 | Physical Interactions |
| IRX5 | SIX1 | 0.014023527 | Shared protein domains |
| TMEM30A | TMEM30B | 1 | Shared protein domains |

# Appendix 6

**GSEA analysis**

**Table S6a. GO of GSEA analysis.**

| Description | enrichmentScore | NES | pvalue | qvalue |
| --- | --- | --- | --- | --- |
| GOMF_EXTRACELLULAR_MATRIX_STRUCTURAL_CONSTITUENT | -0.738744166 | -3.276344481 | 1.00E-10 | 1.21E-08 |
| GOCC_COLLAGEN_CONTAINING_EXTRACELLULAR_MATRIX | -0.645389506 | -3.178880565 | 1.00E-10 | 1.21E-08 |
| GOCC_COLLAGEN_TRIMER | -0.757516376 | -2.951089362 | 1.00E-10 | 1.21E-08 |
| GOBP_COLLAGEN_FIBRIL_ORGANIZATION | -0.760001531 | -2.902456244 | 1.00E-10 | 1.21E-08 |
| GOBP_EXTERNAL_ENCAPSULATING_STRUCTURE_ORGANIZATION | -0.598051436 | -2.811652257 | 1.00E-10 | 1.21E-08 |
| GOCC_BASEMENT_MEMBRANE | -0.685336557 | -2.723595256 | 1.00E-10 | 1.21E-08 |
| GOBP_COLLAGEN_METABOLIC_PROCESS | -0.669654486 | -2.668283414 | 1.00E-10 | 1.21E-08 |
| GOBP_DETECTION_OF_CHEMICAL_STIMULUS_INVOLVED_IN_SENSORY_PERCEPTION_OF_TASTE | 0.861681868 | 2.642360119 | 1.00E-10 | 1.21E-08 |
| GOBP_RETINA_HOMEOSTASIS | 0.782411163 | 2.622534364 | 1.00E-10 | 1.21E-08 |
| GOCC_ENDOPLASMIC_RETICULUM_PROTEIN_CONTAINING_COMPLEX | 0.71961572 | 2.621604187 | 1.00E-10 | 1.21E-08 |
| GOBP_SENSORY_PERCEPTION_OF_BITTER_TASTE | 0.861478227 | 2.609051348 | 1.00E-10 | 1.21E-08 |
| GOBP_SENSORY_PERCEPTION_OF_TASTE | 0.782923994 | 2.557824406 | 1.00E-10 | 1.21E-08 |
| GOMF_CYTOKINE_BINDING | -0.585594664 | -2.553795274 | 1.00E-10 | 1.21E-08 |
| GOCC_SPLICEOSOMAL_COMPLEX | 0.641042236 | 2.474233442 | 1.00E-10 | 1.21E-08 |
| GOBP_ENDOPLASMIC_RETICULUM_TO_GOLGI_VESICLE_MEDIATED_TRANSPORT | 0.667957504 | 2.468463117 | 1.00E-10 | 1.21E-08 |
| GOBP_CYTOPLASMIC_TRANSLATION | 0.646161836 | 2.394730307 | 1.00E-10 | 1.21E-08 |
| GOMF_GROWTH_FACTOR_BINDING | -0.560073047 | -2.392805594 | 1.00E-10 | 1.21E-08 |
| GOBP_PROTEIN_RNA_COMPLEX_ORGANIZATION | 0.604681316 | 2.337544461 | 1.00E-10 | 1.21E-08 |
| GOBP_POSITIVE_REGULATION_OF_VASCULATURE_DEVELOPMENT | -0.530171515 | -2.335654337 | 1.00E-10 | 1.21E-08 |
| GOMF_INTEGRIN_BINDING | -0.535454687 | -2.335133386 | 1.00E-10 | 1.21E-08 |
| GOBP_MRNA_PROCESSING | 0.560418641 | 2.329093769 | 1.00E-10 | 1.21E-08 |
| GOBP_RNA_SPLICING | 0.56228318 | 2.326276619 | 1.00E-10 | 1.21E-08 |
| GOBP_RNA_SPLICING_VIA_TRANSESTERIFICATION_REACTIONS | 0.579102489 | 2.323413927 | 1.00E-10 | 1.21E-08 |
| GOCC_RIBOSOME | 0.59135757 | 2.312044117 | 1.00E-10 | 1.21E-08 |
| GOBP_GOLGI_VESICLE_TRANSPORT | 0.573877495 | 2.304935107 | 1.00E-10 | 1.21E-08 |
| GOMF_GLYCOSAMINOGLYCAN_BINDING | -0.491276784 | -2.268096397 | 1.00E-10 | 1.21E-08 |
| GOBP_PHAGOCYTOSIS | -0.490101702 | -2.245849283 | 1.00E-10 | 1.21E-08 |
| GOBP_RIBONUCLEOPROTEIN_COMPLEX_BIOGENESIS | 0.540149822 | 2.238432308 | 1.00E-10 | 1.21E-08 |
| GOBP_REGULATION_OF_VASCULATURE_DEVELOPMENT | -0.475988781 | -2.235401957 | 1.00E-10 | 1.21E-08 |
| GOBP_HEMOSTASIS | -0.46731067 | -2.149299409 | 1.00E-10 | 1.21E-08 |
| GOBP_TAXIS | -0.431108946 | -2.12624584 | 1.00E-10 | 1.21E-08 |
| GOBP_CONNECTIVE_TISSUE_DEVELOPMENT | -0.454305267 | -2.120212873 | 1.00E-10 | 1.21E-08 |
| GOBP_SKELETAL_SYSTEM_DEVELOPMENT | -0.414531927 | -2.076884213 | 1.00E-10 | 1.21E-08 |
| GOBP_CELL_SUBSTRATE_ADHESION | -0.425242386 | -2.044157669 | 1.00E-10 | 1.21E-08 |
| GOCC_ENDOPLASMIC_RETICULUM_LUMEN | -0.433669697 | -2.039331161 | 1.00E-10 | 1.21E-08 |
| GOBP_LEUKOCYTE_MIGRATION | -0.408521062 | -1.999242687 | 1.00E-10 | 1.21E-08 |
| GOMF_SIGNALING_RECEPTOR_REGULATOR_ACTIVITY | -0.398347877 | -1.998065496 | 1.00E-10 | 1.21E-08 |
| GOBP_REGULATION_OF_AMIDE_METABOLIC_PROCESS | 0.480488499 | 1.996057372 | 1.00E-10 | 1.21E-08 |
| GOBP_REGULATION_OF_INFLAMMATORY_RESPONSE | -0.406862849 | -1.987135183 | 1.00E-10 | 1.21E-08 |
| GOMF_G_PROTEIN_COUPLED_RECEPTOR_ACTIVITY | -0.380949348 | -1.901836991 | 1.00E-10 | 1.21E-08 |
| GOCC_MITOCHONDRIAL_MATRIX | 0.464780131 | 1.93392754 | 2.90E-10 | 3.41E-08 |
| GOBP_BONE_DEVELOPMENT | -0.467538072 | -2.130030429 | 3.31E-10 | 3.71E-08 |
| GOBP_NEGATIVE_REGULATION_OF_LOCOMOTION | -0.401370871 | -1.927188667 | 3.24E-10 | 3.71E-08 |
| GOBP_TRANSLATIONAL_INITIATION | 0.657855341 | 2.380663629 | 4.03E-10 | 4.37E-08 |
| GOBP_CELL_CHEMOTAXIS | -0.424674921 | -1.995744004 | 4.08E-10 | 4.37E-08 |
| GOMF_CADHERIN_BINDING | 0.507364442 | 2.05393869 | 5.41E-10 | 5.67E-08 |
| GOBP_ENDOTHELIAL_CELL_PROLIFERATION | -0.500423841 | -2.204745736 | 6.50E-10 | 6.66E-08 |
| GOMF_MRNA_BINDING | 0.509350742 | 2.062444454 | 9.88E-10 | 9.72E-08 |
| GOBP_POSITIVE_REGULATION_OF_PROGRAMMED_CELL_DEATH | -0.353613459 | -1.771882544 | 9.81E-10 | 9.72E-08 |
| GOCC_EXTERNAL_SIDE_OF_PLASMA_MEMBRANE | -0.387281514 | -1.880178345 | 1.05E-09 | 1.01E-07 |
| GOCC_RIBOSOMAL_SUBUNIT | 0.59572354 | 2.260132281 | 1.10E-09 | 1.04E-07 |
| GOBP_CARTILAGE_DEVELOPMENT | -0.472812388 | -2.145488138 | 1.12E-09 | 1.04E-07 |
| GOBP_POST_TRANSCRIPTIONAL_REGULATION_OF_GENE_EXPRESSION | 0.447104607 | 1.867836912 | 1.66E-09 | 1.51E-07 |
| GOMF_EXTRACELLULAR_MATRIX_STRUCTURAL_CONSTITUENT_CONFERRING_TENSILE_STRENGTH | -0.720541526 | -2.506039997 | 1.99E-09 | 1.78E-07 |
| GOBP_RESPONSE_TO_TYPE_II_INTERFERON | -0.523384588 | -2.230990419 | 2.05E-09 | 1.79E-07 |
| GOBP_REGULATION_OF_MRNA_METABOLIC_PROCESS | 0.505172035 | 2.020680203 | 2.26E-09 | 1.95E-07 |
| GOBP_ENDODERMAL_CELL_DIFFERENTIATION | -0.71313287 | -2.505783417 | 2.59E-09 | 2.19E-07 |
| GOMF_RIBONUCLEOPROTEIN_COMPLEX_BINDING | 0.591807941 | 2.225115588 | 2.74E-09 | 2.28E-07 |
| GOBP_NEGATIVE_REGULATION_OF_CELL_ACTIVATION | -0.449412409 | -2.043032525 | 3.10E-09 | 2.51E-07 |
| GOBP_POSITIVE_REGULATION_OF_CELL_ADHESION | -0.352696167 | -1.762288113 | 3.13E-09 | 2.51E-07 |
| GOBP_MYELOID_LEUKOCYTE_MIGRATION | -0.442093741 | -2.041031153 | 3.25E-09 | 2.57E-07 |
| GOBP_ESTABLISHMENT_OF_PROTEIN_LOCALIZATION_TO_MEMBRANE | 0.526559622 | 2.090519129 | 3.81E-09 | 2.97E-07 |
| GOBP_RESPONSE_TO_ENDOPLASMIC_RETICULUM_STRESS | 0.524273127 | 2.081441405 | 4.92E-09 | 3.76E-07 |
| GOBP_INTEGRIN_MEDIATED_SIGNALING_PATHWAY | -0.546223989 | -2.24925561 | 5.32E-09 | 4.00E-07 |
| GOBP_BASEMENT_MEMBRANE_ORGANIZATION | -0.750200265 | -2.477791502 | 6.02E-09 | 4.47E-07 |
| GOCC_MITOCHONDRIAL_PROTEIN_CONTAINING_COMPLEX | 0.513600753 | 2.043987282 | 6.22E-09 | 4.54E-07 |
| GOMF_TRANSCRIPTION_COREGULATOR_ACTIVITY | 0.443789532 | 1.852293017 | 6.63E-09 | 4.77E-07 |
| GOBP_POSITIVE_REGULATION_OF_ERK1_AND_ERK2_CASCADE | -0.451016995 | -2.042710634 | 7.06E-09 | 5.01E-07 |
| GOBP_COTRANSLATIONAL_PROTEIN_TARGETING_TO_MEMBRANE | 0.890887053 | 2.385793212 | 8.41E-09 | 5.87E-07 |
| GOMF_TRANSLATION_REGULATOR_ACTIVITY_NUCLEIC_ACID_BINDING | 0.654057655 | 2.311748695 | 9.26E-09 | 6.29E-07 |
| GOBP_PROTEIN_TARGETING | 0.489129924 | 1.980661615 | 9.14E-09 | 6.29E-07 |
| GOBP_ESTABLISHMENT_OF_PROTEIN_LOCALIZATION_TO_ORGANELLE | 0.447482815 | 1.852680989 | 1.01E-08 | 6.76E-07 |
| GOBP_ENDOTHELIAL_CELL_MIGRATION | -0.437759422 | -1.995176419 | 1.13E-08 | 7.49E-07 |
| GOMF_CYTOKINE_ACTIVITY | -0.43176913 | -1.978545244 | 1.22E-08 | 7.96E-07 |
| GOBP_SMOOTH_MUSCLE_CELL_MIGRATION | -0.622225827 | -2.386447257 | 1.53E-08 | 9.80E-07 |
| GOCC_PLATELET_ALPHA_GRANULE | -0.573468893 | -2.279022094 | 1.57E-08 | 9.97E-07 |
| GOBP_RIBOSOME_BIOGENESIS | 0.490833767 | 1.976964328 | 1.72E-08 | 1.08E-06 |
| GOMF_TRANSLATION_FACTOR_ACTIVITY_RNA_BINDING | 0.693092721 | 2.358420847 | 1.85E-08 | 1.14E-06 |
| GOBP_POSITIVE_REGULATION_OF_CELL_ACTIVATION | -0.370473163 | -1.802713139 | 1.90E-08 | 1.16E-06 |
| GOCC_CATALYTIC_STEP_2_SPLICEOSOME | 0.660522521 | 2.285412796 | 2.46E-08 | 1.48E-06 |
| GOBP_ENDODERM_FORMATION | -0.653278789 | -2.407076832 | 2.64E-08 | 1.55E-06 |
| GOCC_NUCLEAR_SPECK | 0.459120595 | 1.895519893 | 2.64E-08 | 1.55E-06 |
| GOBP_NEGATIVE_REGULATION_OF_IMMUNE_SYSTEM_PROCESS | -0.337326205 | -1.683869084 | 3.22E-08 | 1.87E-06 |
| GOBP_OSSIFICATION | -0.361912451 | -1.765775906 | 3.29E-08 | 1.89E-06 |
| GOCC_CYTOSOLIC_RIBOSOME | 0.648272523 | 2.284154859 | 3.62E-08 | 2.05E-06 |
| GOMF_TRANSLATION_REGULATOR_ACTIVITY | 0.599111275 | 2.203494672 | 3.78E-08 | 2.11E-06 |
| GOBP_SENSORY_PERCEPTION_OF_CHEMICAL_STIMULUS | 0.552210344 | 2.116142456 | 3.82E-08 | 2.11E-06 |
| GOBP_RESPONSE_TO_AMYLOID_BETA | -0.668318496 | -2.372798735 | 4.45E-08 | 2.44E-06 |
| GOBP_REGULATION_OF_CELLULAR_RESPONSE_TO_GROWTH_FACTOR_STIMULUS | -0.384221167 | -1.810704051 | 4.51E-08 | 2.44E-06 |
| GOMF_PLATELET_DERIVED_GROWTH_FACTOR_BINDING | -0.930373763 | -2.295994601 | 4.74E-08 | 2.49E-06 |
| GOBP_TISSUE_REMODELING | -0.464148747 | -2.069195246 | 4.75E-08 | 2.49E-06 |
| GOBP_CYTOKINE_MEDIATED_SIGNALING_PATHWAY | -0.337106373 | -1.687903359 | 4.69E-08 | 2.49E-06 |
| GOBP_RESPONSE_TO_TRANSFORMING_GROWTH_FACTOR_BETA | -0.40195399 | -1.87255836 | 4.82E-08 | 2.50E-06 |
| GOBP_PHAGOCYTOSIS_RECOGNITION | -0.80393417 | -2.43981646 | 4.94E-08 | 2.52E-06 |
| GOBP_MUSCLE_CELL_MIGRATION | -0.571349654 | -2.264053274 | 4.97E-08 | 2.52E-06 |
| GOBP_RESPONSE_TO_TUMOR_NECROSIS_FACTOR | -0.410787641 | -1.88895399 | 5.77E-08 | 2.89E-06 |
| GOMF_COLLAGEN_BINDING | -0.621680863 | -2.37420772 | 6.04E-08 | 3.00E-06 |
| GOMF_STRUCTURAL_CONSTITUENT_OF_RIBOSOME | 0.572651071 | 2.141778233 | 6.09E-08 | 3.00E-06 |
| GOBP_REGULATION_OF_CHEMOTAXIS | -0.420837353 | -1.928451309 | 6.27E-08 | 3.05E-06 |
| GOBP_EXTRACELLULAR_MATRIX_DISASSEMBLY | -0.635926526 | -2.410117303 | 6.53E-08 | 3.15E-06 |
| GOBP_POSITIVE_REGULATION_OF_REACTIVE_OXYGEN_SPECIES_METABOLIC_PROCESS | -0.615505827 | -2.351148962 | 7.95E-08 | 3.79E-06 |
| GOBP_INTERLEUKIN_6_PRODUCTION | -0.479374967 | -2.089744401 | 8.58E-08 | 4.05E-06 |
| GOBP_MYELOID_LEUKOCYTE_ACTIVATION | -0.420932421 | -1.935992182 | 8.86E-08 | 4.15E-06 |
| GOMF_HEPARIN_BINDING | -0.466237809 | -2.062973831 | 1.09E-07 | 5.01E-06 |
| GOBP_REGULATION_OF_BLOOD_PRESSURE | -0.445528031 | -1.991995096 | 1.08E-07 | 5.01E-06 |
| GOBP_MUSCLE_CELL_PROLIFERATION | -0.421684888 | -1.921130482 | 1.21E-07 | 5.52E-06 |
| GOMF_CCR_CHEMOKINE_RECEPTOR_BINDING | -0.73687827 | -2.386782809 | 1.25E-07 | 5.61E-06 |
| GOMF_ORGANIC_ACID_BINDING | -0.435679384 | -1.973834879 | 1.26E-07 | 5.61E-06 |
| GOBP_NEGATIVE_REGULATION_OF_INFLAMMATORY_RESPONSE | -0.456778837 | -2.012456463 | 1.29E-07 | 5.73E-06 |
| GOMF_CATALYTIC_ACTIVITY_ACTING_ON_RNA | 0.456373221 | 1.865728428 | 1.37E-07 | 6.01E-06 |
| GOBP_POSITIVE_REGULATION_OF_TUMOR_NECROSIS_FACTOR_SUPERFAMILY_CYTOKINE_PRODUCTION | -0.527811249 | -2.153055846 | 1.39E-07 | 6.01E-06 |
| GOBP_ASTROCYTE_DEVELOPMENT | -0.693665088 | -2.357194425 | 1.49E-07 | 6.39E-06 |
| GOBP_ESTABLISHMENT_OF_PROTEIN_LOCALIZATION_TO_ENDOPLASMIC_RETICULUM | 0.775874046 | 2.379229172 | 1.54E-07 | 6.56E-06 |
| GOMF_METALLOENDOPEPTIDASE_ACTIVITY | -0.515583618 | -2.123083876 | 1.58E-07 | 6.67E-06 |
| GOBP_ENDODERM_DEVELOPMENT | -0.563538295 | -2.211822307 | 1.63E-07 | 6.78E-06 |
| GOBP_CELLULAR_RESPONSE_TO_TYPE_II_INTERFERON | -0.513008191 | -2.116144937 | 1.63E-07 | 6.78E-06 |
| GOBP_TRANSMEMBRANE_RECEPTOR_PROTEIN_SERINE_THREONINE_KINASE_SIGNALING_PATHWAY | -0.359252943 | -1.743453401 | 1.80E-07 | 7.40E-06 |
| GOBP_GASTRULATION | -0.438942124 | -1.963134382 | 1.85E-07 | 7.54E-06 |
| GOBP_REGULATION_OF_INTEGRIN_MEDIATED_SIGNALING_PATHWAY | -0.805655833 | -2.341173615 | 1.86E-07 | 7.54E-06 |
| GOMF_CYTOKINE_RECEPTOR_BINDING | -0.408168181 | -1.878558477 | 1.91E-07 | 7.67E-06 |
| GOBP_TUMOR_NECROSIS_FACTOR_SUPERFAMILY_CYTOKINE_PRODUCTION | -0.460642055 | -2.038214161 | 1.97E-07 | 7.86E-06 |
| GOBP_PROTEIN_LOCALIZATION_TO_ENDOPLASMIC_RETICULUM | 0.693581389 | 2.305202974 | 2.10E-07 | 8.31E-06 |
| GOBP_SMOOTH_MUSCLE_CELL_PROLIFERATION | -0.479114896 | -2.075293969 | 2.20E-07 | 8.56E-06 |
| GOBP_TISSUE_HOMEOSTASIS | 0.482907913 | 1.928577154 | 2.20E-07 | 8.56E-06 |
| GOBP_POSITIVE_REGULATION_OF_CYTOKINE_PRODUCTION | -0.333131976 | -1.662143745 | 2.36E-07 | 9.10E-06 |
| GOBP_COLLAGEN_CATABOLIC_PROCESS | -0.674781663 | -2.312874375 | 2.41E-07 | 9.23E-06 |
| GOBP_POSITIVE_REGULATION_OF_MAPK_CASCADE | -0.332358126 | -1.647418528 | 2.63E-07 | 1.00E-05 |
| GOBP_MONONUCLEAR_CELL_MIGRATION | -0.42913777 | -1.944198257 | 2.82E-07 | 1.06E-05 |
| GOBP_REGULATION_OF_COAGULATION | -0.565110072 | -2.18474072 | 2.97E-07 | 1.11E-05 |
| GOBP_AMEBOIDAL_TYPE_CELL_MIGRATION | -0.335115186 | -1.655336879 | 3.04E-07 | 1.13E-05 |
| GOCC_U2_TYPE_SPLICEOSOMAL_COMPLEX | 0.637379855 | 2.237503875 | 3.11E-07 | 1.14E-05 |
| GOBP_REGULATION_OF_ENDOCYTOSIS | -0.382172424 | -1.790728549 | 3.14E-07 | 1.15E-05 |
| GOBP_EOSINOPHIL_MIGRATION | -0.757055331 | -2.301959616 | 3.35E-07 | 1.22E-05 |
| GOBP_REGULATION_OF_RNA_SPLICING | 0.539237061 | 2.061493599 | 3.63E-07 | 1.30E-05 |
| GOBP_POSITIVE_REGULATION_OF_SMOOTH_MUSCLE_CELL_MIGRATION | -0.689302015 | -2.318967186 | 3.76E-07 | 1.34E-05 |
| GOBP_NEGATIVE_REGULATION_OF_AMIDE_METABOLIC_PROCESS | 0.504724588 | 1.971192234 | 3.90E-07 | 1.38E-05 |
| GOBP_ADENYLATE_CYCLASE_MODULATING_G_PROTEIN_COUPLED_RECEPTOR_SIGNALING_PATHWAY | -0.405529695 | -1.858871582 | 4.02E-07 | 1.41E-05 |
| GOBP_LEUKOCYTE_CHEMOTAXIS | -0.403811778 | -1.851610591 | 4.18E-07 | 1.46E-05 |
| GOBP_CHEMOKINE_PRODUCTION | -0.515706415 | -2.068278102 | 4.37E-07 | 1.51E-05 |
| GOCC_BASAL_PART_OF_CELL | 0.468918378 | 1.893697013 | 4.43E-07 | 1.53E-05 |
| GOBP_POSITIVE_REGULATION_OF_INFLAMMATORY_RESPONSE | -0.460679744 | -1.990614493 | 4.47E-07 | 1.53E-05 |
| GOBP_SRP_DEPENDENT_COTRANSLATIONAL_PROTEIN_TARGETING_TO_MEMBRANE | 0.890832317 | 2.245904816 | 4.55E-07 | 1.54E-05 |
| GOBP_NEGATIVE_REGULATION_OF_RESPONSE_TO_EXTERNAL_STIMULUS | -0.339369778 | -1.672042358 | 4.61E-07 | 1.56E-05 |
| GOCC_COMPLEX_OF_COLLAGEN_TRIMERS | -0.809943341 | -2.387929432 | 4.96E-07 | 1.66E-05 |
| GOBP_BRANCHING_INVOLVED_IN_BLOOD_VESSEL_MORPHOGENESIS | -0.689866449 | -2.301152282 | 5.05E-07 | 1.67E-05 |
| GOBP_VASCULAR_PROCESS_IN_CIRCULATORY_SYSTEM | -0.389494199 | -1.820133146 | 5.04E-07 | 1.67E-05 |
| GOBP_SECRETION_BY_TISSUE | 0.732479498 | 2.279666267 | 5.12E-07 | 1.68E-05 |
| GOBP_CELLULAR_RESPONSE_TO_TOPOLOGICALLY_INCORRECT_PROTEIN | 0.60202086 | 2.15183747 | 5.31E-07 | 1.73E-05 |
| GOMF_SULFUR_COMPOUND_BINDING | -0.386795118 | -1.787471804 | 5.36E-07 | 1.73E-05 |
| GOBP_GRANULOCYTE_MIGRATION | -0.46194388 | -2.013756875 | 5.71E-07 | 1.84E-05 |
| GOCC_LARGE_RIBOSOMAL_SUBUNIT | 0.612221802 | 2.182312347 | 5.78E-07 | 1.85E-05 |
| GOCC_ENDOPLASMIC_RETICULUM_GOLGI_INTERMEDIATE_COMPARTMENT | 0.570774895 | 2.099275195 | 6.05E-07 | 1.91E-05 |
| GOBP_MITOCHONDRIAL_GENE_EXPRESSION | 0.537239145 | 2.030989702 | 6.10E-07 | 1.91E-05 |
| GOMF_IMMUNE_RECEPTOR_ACTIVITY | -0.461725095 | -1.988486127 | 6.07E-07 | 1.91E-05 |
| GOBP_ERK1_AND_ERK2_CASCADE | -0.36597717 | -1.72058869 | 6.24E-07 | 1.94E-05 |
| GOMF_TRANSFORMING_GROWTH_FACTOR_BETA_BINDING | -0.768910159 | -2.329898199 | 6.50E-07 | 2.01E-05 |
| GOMF_TRANSLATION_INITIATION_FACTOR_ACTIVITY | 0.736558097 | 2.341675992 | 6.56E-07 | 2.01E-05 |
| GOMF_FIBRONECTIN_BINDING | -0.731040529 | -2.335612101 | 6.70E-07 | 2.05E-05 |
| GOBP_NEUROINFLAMMATORY_RESPONSE | -0.579046049 | -2.226120165 | 7.05E-07 | 2.11E-05 |
| GOBP_ENDOPLASMIC_RETICULUM_UNFOLDED_PROTEIN_RESPONSE | 0.648256694 | 2.179190186 | 7.02E-07 | 2.11E-05 |
| GOBP_REGULATION_OF_TUBE_SIZE | -0.45933152 | -1.99613287 | 6.96E-07 | 2.11E-05 |
| GOMF_HORMONE_ACTIVITY | -0.493514606 | -2.040251315 | 7.27E-07 | 2.16E-05 |
| GOBP_RESPONSE_TO_MOLECULE_OF_BACTERIAL_ORIGIN | -0.357893289 | -1.720426164 | 7.88E-07 | 2.33E-05 |
| GOBP_GOLGI_ORGANIZATION | 0.559660431 | 2.074148799 | 8.50E-07 | 2.50E-05 |
| GOBP_T_CELL_ACTIVATION_VIA_T_CELL_RECEPTOR_CONTACT_WITH_ANTIGEN_BOUND_TO_MHC_MOLECULE_ON_ANTIGEN_PRESENTING_CELL | -0.912130301 | -2.18333581 | 8.80E-07 | 2.54E-05 |
| GOBP_PROTEASOMAL_PROTEIN_CATABOLIC_PROCESS | 0.40750339 | 1.705135112 | 8.79E-07 | 2.54E-05 |
| GOCC_RECEPTOR_COMPLEX | -0.338490346 | -1.656338562 | 8.80E-07 | 2.54E-05 |
| GOBP_NEUTROPHIL_MIGRATION | -0.493835966 | -2.07110944 | 9.01E-07 | 2.59E-05 |
| GOBP_REGULATION_OF_LYMPHOCYTE_ACTIVATION | -0.321695638 | -1.606566461 | 9.27E-07 | 2.64E-05 |
| GOMF_STRUCTURAL_MOLECULE_ACTIVITY_CONFERRING_ELASTICITY | -0.876641839 | -2.215608664 | 9.48E-07 | 2.69E-05 |
| GOBP_REGULATION_OF_PHAGOCYTOSIS | -0.5183563 | -2.077991041 | 9.59E-07 | 2.70E-05 |
| GOBP_DETECTION_OF_STIMULUS_INVOLVED_IN_SENSORY_PERCEPTION | 0.509392813 | 1.974729394 | 9.83E-07 | 2.75E-05 |
| GOMF_METALLOPEPTIDASE_ACTIVITY | -0.42884433 | -1.912092361 | 1.07E-06 | 2.96E-05 |
| GOBP_MACROAUTOPHAGY | 0.452591829 | 1.831939083 | 1.07E-06 | 2.96E-05 |
| GOBP_CELLULAR_RESPONSE_TO_AMYLOID_BETA | -0.667511588 | -2.26832029 | 1.11E-06 | 3.05E-05 |
| GOBP_REGULATION_OF_LEUKOCYTE_MIGRATION | -0.395770969 | -1.814139425 | 1.12E-06 | 3.05E-05 |
| GOBP_CHONDROCYTE_DIFFERENTIATION | -0.499661725 | -2.059455702 | 1.13E-06 | 3.07E-05 |
| GOBP_APOPTOTIC_CELL_CLEARANCE | -0.641928911 | -2.2227576 | 1.27E-06 | 3.44E-05 |
| GOBP_POSITIVE_REGULATION_OF_INTEGRIN_MEDIATED_SIGNALING_PATHWAY | -0.872807214 | -2.205917104 | 1.30E-06 | 3.51E-05 |
| GOBP_ADENYLATE_CYCLASE_ACTIVATING_G_PROTEIN_COUPLED_RECEPTOR_SIGNALING_PATHWAY | -0.466214462 | -1.984917275 | 1.37E-06 | 3.65E-05 |
| GOBP_WOUND_HEALING | -0.337524864 | -1.64923694 | 1.37E-06 | 3.65E-05 |
| GOBP_VASCULOGENESIS | -0.534765851 | -2.119084817 | 1.47E-06 | 3.88E-05 |
| GOCC_PROTEIN_COMPLEX_INVOLVED_IN_CELL_MATRIX_ADHESION | -0.82721137 | -2.26489894 | 1.48E-06 | 3.89E-05 |
| GOCC_LATERAL_PLASMA_MEMBRANE | 0.663671476 | 2.199167436 | 1.61E-06 | 4.23E-05 |
| GOBP_HEART_MORPHOGENESIS | -0.375801311 | -1.73139559 | 1.71E-06 | 4.45E-05 |
| GOBP_LEUKOCYTE_MEDIATED_IMMUNITY | -0.338345627 | -1.648022624 | 1.72E-06 | 4.47E-05 |
| GOBP_FORMATION_OF_PRIMARY_GERM_LAYER | -0.485968075 | -2.038112118 | 1.81E-06 | 4.66E-05 |
| GOMF_PROTEASE_BINDING | -0.459228312 | -1.963527702 | 1.82E-06 | 4.66E-05 |
| GOBP_POSITIVE_REGULATION_OF_LYMPHOCYTE_ACTIVATION | -0.348429069 | -1.660234819 | 1.88E-06 | 4.80E-05 |
| GOBP_REGULATION_OF_CELL_SUBSTRATE_ADHESION | -0.40606469 | -1.855484359 | 1.92E-06 | 4.88E-05 |
| GOBP_REGULATION_OF_IMMUNE_EFFECTOR_PROCESS | -0.337443742 | -1.643437647 | 1.94E-06 | 4.89E-05 |
| GOMF_RIBOSOME_BINDING | 0.601432688 | 2.120529989 | 2.06E-06 | 5.13E-05 |
| GOMF_MONOCARBOXYLIC_ACID_BINDING | -0.545399173 | -2.119396791 | 2.05E-06 | 5.13E-05 |
| GOBP_CELLULAR_RESPONSE_TO_ZINC_ION | -0.789128819 | -2.326562658 | 2.16E-06 | 5.37E-05 |
| GOBP_TRANSFORMING_GROWTH_FACTOR_BETA_RECEPTOR_SIGNALING_PATHWAY | -0.394112398 | -1.795514525 | 2.24E-06 | 5.54E-05 |
| GOBP_SALIVA_SECRETION | 0.892594454 | 2.11689725 | 2.27E-06 | 5.57E-05 |
| GOBP_CELL_MATRIX_ADHESION | -0.393215124 | -1.815371364 | 2.37E-06 | 5.80E-05 |
| GOBP_RRNA_METABOLIC_PROCESS | 0.471862125 | 1.86622034 | 2.51E-06 | 6.07E-05 |
| GOBP_NEGATIVE_REGULATION_OF_CELL_ADHESION | -0.364990466 | -1.708446558 | 2.50E-06 | 6.07E-05 |

**Table S6b. KEGG of GSEA analysis.**

| Description | enrichmentScore | NES | pvalue | qvalue |
| --- | --- | --- | --- | --- |
| KEGG_ECM_RECEPTOR_INTERACTION | -0.670993776 | -2.641663963 | 1.00E-10 | 4.60E-09 |
| KEGG_FOCAL_ADHESION | -0.4937352 | -2.257189407 | 1.00E-10 | 4.60E-09 |
| KEGG_NEUROACTIVE_LIGAND_RECEPTOR_INTERACTION | -0.445663993 | -2.076836928 | 1.00E-10 | 4.60E-09 |
| KEGG_SPLICEOSOME | 0.623763362 | 2.278186331 | 8.19E-09 | 2.82E-07 |
| KEGG_COMPLEMENT_AND_COAGULATION_CASCADES | -0.619854644 | -2.359698795 | 1.14E-08 | 3.14E-07 |
| KEGG_CYTOKINE_CYTOKINE_RECEPTOR_INTERACTION | -0.406650935 | -1.906704321 | 3.33E-08 | 7.66E-07 |
| KEGG_SYSTEMIC_LUPUS_ERYTHEMATOSUS | -0.641681746 | -2.290103365 | 1.47E-07 | 2.89E-06 |
| KEGG_PROTEIN_EXPORT | 0.858749285 | 2.30083351 | 1.85E-07 | 3.18E-06 |
| KEGG_RIBOSOME | 0.64288624 | 2.161641183 | 1.80E-06 | 2.76E-05 |
| KEGG_LEISHMANIA_INFECTION | -0.507016016 | -1.930138128 | 5.12E-05 | 0.000705379 |
| KEGG_PPAR_SIGNALING_PATHWAY | -0.521995112 | -1.967831163 | 6.33E-05 | 0.000794072 |
| KEGG_RENIN_ANGIOTENSIN_SYSTEM | -0.740000143 | -2.02345738 | 0.000141789 | 0.001629326 |
| KEGG_ASTHMA | -0.658358443 | -2.022838532 | 0.000256511 | 0.002720883 |
| KEGG_CHEMOKINE_SIGNALING_PATHWAY | -0.357984132 | -1.605889081 | 0.000367588 | 0.003620607 |
| KEGG_VIBRIO_CHOLERAE_INFECTION | 0.590425337 | 1.867347081 | 0.000733486 | 0.006407054 |
| KEGG_DILATED_CARDIOMYOPATHY | -0.435819053 | -1.751897227 | 0.000743414 | 0.006407054 |
| KEGG_UBIQUITIN_MEDIATED_PROTEOLYSIS | 0.475086177 | 1.74739296 | 0.000807451 | 0.006549599 |
| KEGG_OXIDATIVE_PHOSPHORYLATION | 0.509334538 | 1.805456587 | 0.001040296 | 0.007918766 |
| KEGG_HYPERTROPHIC_CARDIOMYOPATHY_HCM | -0.43854507 | -1.726526756 | 0.001091097 | 0.007918766 |
| KEGG_N_GLYCAN_BIOSYNTHESIS | 0.600676646 | 1.883232812 | 0.001406539 | 0.009697713 |
| KEGG_PATHWAYS_IN_CANCER | -0.287900017 | -1.361477917 | 0.001944486 | 0.012768305 |
| KEGG_TYPE_I_DIABETES_MELLITUS | -0.528575654 | -1.802778658 | 0.002650424 | 0.016612705 |
| KEGG_ALLOGRAFT_REJECTION | -0.535426047 | -1.757731119 | 0.003321645 | 0.019914666 |
| KEGG_GLYCOSAMINOGLYCAN_BIOSYNTHESIS_CHONDROITIN_SULFATE | -0.608228608 | -1.799549676 | 0.003824782 | 0.021975719 |
| KEGG_TOLL_LIKE_RECEPTOR_SIGNALING_PATHWAY | -0.37275326 | -1.530475153 | 0.00619187 | 0.034153049 |
| KEGG_PROTEASOME | 0.560507553 | 1.734939304 | 0.006825296 | 0.036198937 |
| KEGG_HEMATOPOIETIC_CELL_LINEAGE | -0.38980164 | -1.538376152 | 0.008470204 | 0.043259131 |
| KEGG_GLYCOSYLPHOSPHATIDYLINOSITOL_GPI_ANCHOR_BIOSYNTHESIS | 0.626613268 | 1.71916885 | 0.011063092 | 0.046228552 |
| KEGG_O_GLYCAN_BIOSYNTHESIS | 0.604836804 | 1.717574771 | 0.010037033 | 0.046228552 |
| KEGG_LONG_TERM_POTENTIATION | 0.487351952 | 1.630226696 | 0.010983291 | 0.046228552 |
| KEGG_ABC_TRANSPORTERS | -0.470258795 | -1.62133296 | 0.010876139 | 0.046228552 |
| KEGG_ADIPOCYTOKINE_SIGNALING_PATHWAY | -0.416049037 | -1.562452458 | 0.009503153 | 0.046228552 |
| KEGG_JAK_STAT_SIGNALING_PATHWAY | -0.319956913 | -1.402439369 | 0.010709806 | 0.046228552 |
| KEGG_BUTANOATE_METABOLISM | 0.570506939 | 1.681271216 | 0.014551919 | 0.057332371 |
| KEGG_SMALL_CELL_LUNG_CANCER | -0.375016821 | -1.496561791 | 0.014216904 | 0.057332371 |
| KEGG_CELL_ADHESION_MOLECULES_CAMS | -0.339833003 | -1.43274577 | 0.015231687 | 0.058343595 |
| KEGG_AXON_GUIDANCE | -0.32660175 | -1.385185602 | 0.016412197 | 0.061166368 |
| KEGG_RIBOFLAVIN_METABOLISM | 0.678317069 | 1.659764493 | 0.020160881 | 0.064939078 |
| KEGG_SNARE_INTERACTIONS_IN_VESICULAR_TRANSPORT | 0.547356798 | 1.655217782 | 0.018492154 | 0.064939078 |
| KEGG_RNA_DEGRADATION | 0.499676887 | 1.580335597 | 0.019269435 | 0.064939078 |
| KEGG_ENDOMETRIAL_CANCER | 0.494512473 | 1.571814083 | 0.019860435 | 0.064939078 |
| KEGG_PHOSPHATIDYLINOSITOL_SIGNALING_SYSTEM | 0.447360663 | 1.536174417 | 0.020250087 | 0.064939078 |
| KEGG_NATURAL_KILLER_CELL_MEDIATED_CYTOTOXICITY | -0.321049967 | -1.361639341 | 0.020030566 | 0.064939078 |
| KEGG_VASOPRESSIN_REGULATED_WATER_REABSORPTION | 0.509193867 | 1.596417316 | 0.025097755 | 0.078655643 |
| KEGG_INTESTINAL_IMMUNE_NETWORK_FOR_IGA_PRODUCTION | 0.49252921 | 1.544170521 | 0.035136597 | 0.107670039 |
| KEGG_LYSOSOME | 0.396765342 | 1.448076781 | 0.040673212 | 0.121926562 |
| KEGG_WNT_SIGNALING_PATHWAY | 0.372236314 | 1.397390379 | 0.046070461 | 0.135167533 |

# Appendix 7

**Immune Correlation Analysis**

**Table S7. Immune Correlation Analysis.**

| Gene | Cell | cor | pvalue |
| --- | --- | --- | --- |
| ATP2A3 | B cells naive | 0.27280794 | 0.005535565 |
| ATP2A3 | B cells memory | -0.270816644 | 0.005904281 |
| ATP2A3 | Plasma cells | 0.500313824 | 1.31E-07 |
| ATP2A3 | T cells CD8 | -0.187057886 | 0.059759812 |
| ATP2A3 | T cells CD4 naive | 0.161146241 | 0.105655549 |
| ATP2A3 | T cells CD4 memory resting | -0.106649016 | 0.286028898 |
| ATP2A3 | T cells CD4 memory activated | -0.115654905 | 0.247047452 |
| ATP2A3 | T cells follicular helper | 0.097576422 | 0.329233633 |
| ATP2A3 | T cells regulatory (Tregs) | -0.229111647 | 0.02054203 |
| ATP2A3 | T cells gamma delta | -0.043088545 | 0.667188756 |
| ATP2A3 | NK cells resting | 0.029611634 | 0.767656391 |
| ATP2A3 | NK cells activated | -0.034809818 | 0.728340123 |
| ATP2A3 | Monocytes | -0.301391791 | 0.002081806 |
| ATP2A3 | Macrophages M0 | -0.116517097 | 0.243517775 |
| ATP2A3 | Macrophages M1 | -0.083805011 | 0.401735103 |
| ATP2A3 | Macrophages M2 | -0.488585086 | 1.89E-07 |
| ATP2A3 | Dendritic cells resting | -0.12474672 | 0.211577712 |
| ATP2A3 | Dendritic cells activated | -0.003944621 | 0.968613039 |
| ATP2A3 | Mast cells resting | -0.298399676 | 0.002317025 |
| ATP2A3 | Mast cells activated | -0.135935736 | 0.173104451 |
| ATP2A3 | Eosinophils | -0.170664037 | 0.086347934 |
| ATP2A3 | Neutrophils | -0.170664037 | 0.086347934 |
